# Incidence and mortality due to snakebite in the Americas

**DOI:** 10.1371/journal.pntd.0005662

**Published:** 2017-06-21

**Authors:** Jean-Philippe Chippaux

**Affiliations:** 1 CERPAGE, Faculté des Sciences de la Santé, Université d’Abomey-Calavi, Cotonou, Bénin; 2 UMR216, Mère et enfant face aux infections tropicales and PRES Sorbonne Paris Cité, Université Paris Descartes, Faculté de Pharmacie, Paris, France; Universidad de Costa Rica, COSTA RICA

## Abstract

**Background:**

Better knowledge of the epidemiological characteristics of snakebites could help to take measures to improve their management. The incidence and mortality of snakebites in the Americas are most often estimated from medical and scientific literature, which generally lack precision and representativeness.

**Methodology/Principal findings:**

Authors used the notifications of snakebites treated in health centers collected by the Ministries of Health of the American countries to estimate their incidence and mortality. Data were obtained from official reports available on-line at government sites, including those of the Ministry of Health in each country and was sustained by recent literature obtained from PubMed. The average annual incidence is about 57,500 snake bites (6.2 per 100,000 population) and mortality is close to 370 deaths (0.04 per 100,000 population), that is, between one third and half of the previous estimates. The incidence of snakebites is influenced by the abundance of snakes, which is related to (i) climate and altitude, (ii) specific preferences of the snake for environments suitable for their development, and (iii) human population density. Recent literature allowed to notice that the severity of the bites depends mainly on (i) the snake responsible for the bite (species and size) and (ii) accessibility of health care, including availability of antivenoms.

**Conclusions/Significances:**

The main limitation of this study could be the reliability and accuracy of the notifications by national health services. However, the data seemed consistent considering the similarity of the incidences on each side of national boundaries while the sources are distinct. However, snakebite incidence could be underestimated due to the use of traditional medicine by the patients who escaped the reporting of cases. However, gathered data corresponded to the actual use of the health facilities, and therefore to the actual demand for antivenoms, which should make it possible to improve their management.

## Author summary

A better knowledge of snakebites incidence and mortality might improve their management. However, they are difficult to estimate, in particular because most of them are based on extrapolations from scientific and medical publications that are not representative of the epidemiological situation. This study, based on data available on-line at government sites in the Americas—reflecting notifications from health services—sustained by recent publications to provide useful information on snakebites treated in health centers of American countries. On average, nearly 60,000 snake bites are managed every year in health facilities in the Americas and approximately 370 deaths are reported officially. The development of snake populations results from environmental conditions favorable to their feeding and camouflage. Moreover, the activities of human—notably agricultural—explain encounters with the snakes. The literature underlines that the severity of the envenomation depend on the species responsible for the bite and quality of the management of the patient. Without excluding an underestimation of snakebite incidence, due to the frequent use of traditional medicine, this study should enable health authorities to better analyze the epidemiological situation of snakebites, including their frequency, distribution and severity, in order to improve the management of the envenomation.

## Introduction

Snakebite is an important public health issue in the Americas, particularly in inter-tropical America [1; 2]. A better understanding of the epidemiological burden of snakebite, *i*.*e*. incidence, geographical distribution, population at risk, bite circumstances and severity, would improve their management [3] and should be used to urge World Health Organization (WHO) to include definitely snakebites in the list of neglected tropical diseases (NTD) and to convince international agencies and foundations of funding. It would also help the antivenom manufacturers to produce the necessary quantity, and the Health Authorities to supply the health centers according to the declared incidence and the geographical distribution of the envenomations.

However, most data available for the Americas are fragmentary and poorly representative, mainly because they come from the literature, making them incomplete and biased. This is particularly true in Central and South American countries. In recent years, international workshop dedicated to the improvement of antivenoms pointed out that “renewed efforts were required on national and regional basis to improve the epidemiological surveillance system in order to gather a more precise picture of the impact of this health problem” [4] and recommended “to improve the information systems on the epidemiology of snakebite envenomations in the region, that is essential for the design of effective distribution policies and training programs” [5]. As a consequence, most Latin American countries introduced mandatory notification of snakebites during the 2000s.

The bites by opistoglyphic (rear fanged) snakes and those of the families lacking fangs delivering venom (Boidae, Aniilidae in particular) being weakly toxic [6], represent a low demand for health services, although the incidence is far from trivial [7]. The snakes belonging to the Scolecophidia suborder (Typhlopidae and Leptotyphlopidae) are definitely non-toxic and unable to bite. As a consequence, two snake families share responsibility for snake envenomations in the Americas: the Viperidae (including half a dozen genera, the most frequent being *Crotalus*, *Bothrops* and *Agkistrodon*) and the Elapidae of which *Micrurus* is the main genus [8]. The bites of the latter represent less than 1% of the envenomations [9–13].

The symptoms caused by viper bite are mainly hemorrhagic and cytotoxic, the latter sometimes resulting in limb amputation or permanent disability [14; 15]. Some species of *Crotalus* may also produce neurotoxic symptoms similar to envenomation by Elapidae [16], and sometimes associated with acute renal failure [17]. Unlike the neurotoxins of rattlesnake venoms that act on presynaptic receptors (β-neurotoxins), the α-neurotoxins of Elapidae venoms bind to postsynaptic cholinergic receptors [13]. In both cases, paralysis of the cranial nerves can occur, inducing in some cases a potentially fatal respiratory arrest in the absence of specific (antivenom) and/or symptomatic treatment (artificial ventilation).

The aim of this work was to assess the epidemiological burden of snakebite, including the incidence, mortality, population at risk and main explanatory characteristics of their frequency and severity: season, environment, altitude, density of human population, management, etc., in order to provide recent and useful data to improve the management of snakebites in the Americas.

## Methods

A bibliographic search was performed by querying MedLine (PubMed last access 06/11/2016) using the keywords "America AND snake * AND [envenom * OR antiven *]". From a total of 4,514 references, 187 concerned the epidemiology and/or management of snakebites in the Americas.

Furthermore, websites regarding i) the epidemiology of snakebites (using the words “health surveillance”, “surveillance bulletin”, “epidemiology surveillance”, “snakebite envenomation”, “snakebite death”), ii) population demography (using the words “population demography”) and iii) administrative and environmental geography (using the word “map”) were identified using the Google search engine for each of the countries of America and using the official language of each country (English, Spanish, Portuguese, French and Dutch). Access to these websites was made between September 2010 and December 2016. The list of the websites and the last access date to each are mentioned in Table 1. However, a few websites were closed during this period and sometimes replaced by new ones, the use of which was often restricted by a password.

**Table 1:** Web site identification and exploration.

Country	Web site	Last access
Argentina	http://www.msal.gov.ar/saladesituacion/mapa/index_enos.html http://www.msal.gov.ar/index.php/home/boletin-integrado-de-vigilancia	08/11/2016 08/11/2016
Bolivia	http://www.ine.gob.bo/ http://www.sns.gob.bo/NHIS/default.aspx http://estadisticas.minsalud.gob.bo/Reportes_Vigilancia/	09/11/2016 16/07/2012 09/11/2016
Brazil	http://dtr2004.saude.gov.br/sinanweb/tabnet/dh?sinan/animaisp/bases/animaisbr.def http://dtr2004.saude.gov.br/sinanweb/index.php	31/07/2014 31/07/2014
Colombia	http://www.ins.gov.co/boletin-epidemiologico/Paginas/default.aspx http://www.dane.gov.co/ www.bdigital.unal.edu.co/8874/1/598907.2012.pdf	06/11/2016 23/08/2015 06/11/2016
Costa Rica	http://www.ministeriodesalud.go.cr/index.php/biblioteca-de-archivos/centro-de-informacion/material-publicado/boletines-1/boletines-vigilancia-de-la-salud http://www.ministeriodesalud.go.cr/index.php/vigilancia-de-la-salud	08/11/2016 27/10/2013
El Salvador	http://asp.salud.gob.sv/regulacion/pdf/lineamientos/lineamientos_personas_mordidas_por_serpientes.pdf	10/11/2016
Equator	http://www.salud.gob.ec/gaceta-epidemiologica-ecuador-sive-alerta/ http://www.salud.gob.ec/informacion-estadistica-de-produccion-de-salud/ https://public.tableau.com/profile/publish/egresoshospitalariosinec2014/Menu#!/publish-confirm	07/11/2016 07/11/2016 11/11/2016
Guyana	http://www.kaieteurnewsonline.com/2015/04/05/dealing-with-snake-bites/ https://www.google.fr/url?sa=t&rct=j&q=&esrc=s&source=web&cd=1&ved=0ahUKEwjB9eWR06XQAhVFI8AKHS0FBewQFggaMAA&url=http%3A%2F%2Fmedicalcouncil.org.gy%2Fmedcouncil%2Fdocuments%2FCME%2520Presentations%2FManagement%2520of%2520Snake%2520Bites.pptx&usg=AFQjCNGXqJgEoyKCYqM3SpnI72P_kAFkMg&cad=rja http://perspective.usherbrooke.ca/bilan/servlet/BMPagePyramide?codePays=GUY	10/11/2016 27/08/2015 13/11/2016
Honduras	http://www.salud.gob.hn/dgvs_publicaciones.html# https://sites.google.com/site/infosaludhonduras/home/boletin-epidemiologico-de-honduras---2014 http://www.salud.gob.hn/dgvs_bsemanal13.html	10/11/2016 14/11/2016 10/11/2016
Mexico	http://www.epidemiologia.salud.gob.mx/dgae/boletin/intd_boletin.html http://www.epidemiologia.salud.gob.mx/dgae/infoepid/publicaciones_mortalidad.html http://www.epidemiologia.salud.gob.mx/dgae/boletin/intd_historicos.html http://www.inegi.org.mx/est/contenidos/proyectos/ccpv/default.aspx http://www.epidemiologia.salud.gob.mx/anuario/html/anuarios.html	10/11/2016 10/11/2016 10/11/2016 10/11/2016 17/11/2016
Nicaragua	http://www.minsa.gob.ni/index.php/direccion-general-de-vigilancia-de-la-salud-publica/boletin-epidemiologico http://www.minsa.gob.ni/index.php/repository/Descargas-MINSA/Direcci%C3%B3n-General-Vigilancia-de-la-Salud-P%C3%BAblica/Boletines/Boletines-2014/Bolet%C3%ADn-Epidemiol%C3%B3gico-Semana-No.-22/	10/11/2016 10/11/2016
Panama	http://www.minsa.gob.pa/informacion-salud/boletines-semanales http://www.minsa.gob.pa/noticia/minsa-y-se-unen-para-bajar-cifras-de-muertes-por-picaduras-de-serpientes-y-escorpiones	10/11/2016 10/11/2016
Paraguay	http://www.vigisalud.gov.py/index.php?option=com_content&view=category&id=9&Itemid=142	10/11/2016
Peru	http://www.dge.gob.pe/boletin.php http://sisbib.unmsm.edu.pe/BVRevistas/bol_epid/bol_epid.htm http://www.dge.gob.pe/portal/docs/vigilancia/boletines/2013/01.pdf	10/11/2016 10/11/2016 10/11/2016
Uruguay	http://www.msp.gub.uy/ http://www.msp.gub.uy/publicaci%C3%B3n/ofidismo	10/11/2016 10/11/2016
USA	http://webappa.cdc.gov/sasweb/ncipc/nfirates2001.html	10/11/2016
Venezuela	http://www.mpps.gob.ve/index.php?option=com_phocadownload&view=category&id=2:-indicadores-epidemiologicos-de-la-republica-bolivariana-de-venezuela&Itemid=915 http://www.bvs.gob.ve/php/level.php?lang=es&component=35&item=3 http://www.bvs.gob.ve/php/level.php?lang=es&component=35&item=37 http://www.bvs.gob.ve/php/level.php?lang=es&component=35&item=4	10/11/2016 17/11/2016 10/11/2016 17/11/2016

All the data were transferred and analyzed using Excel software. The trend curves and R^2^, the coefficient of determination that is the square of the coefficient of correlation indicates the extent to which the dependent variable is predictable, were calculated through Excel. The comparisons were made using parametric tests (t-test, χ^2^ and Pearson correlation) or non-parametric (Mann-Whitney), depending on the distribution of studied variables and number of cases/groups. The significance level was equal to 0.05 and the means were expressed using a 95% IC. Statistical analyzes were performed using the BiostatTGV online software (http://marne.u707.jussieu.fr/biostatgv/).

Topographic, physical and political maps were taken from the World Atlas of Wikimedia (https://commons.wikimedia.org/wiki/Atlas_of_the_world) and drawn on the basis of the data obtained in this study.

## Results

The average incidence is about 57,500 snakebites a year (6.34 per 100,000 population), resulting in almost 370 deaths (0.037 per 100,000 population), with a case fatality rate below 0.6% (Table 2). However, there are wide variations across countries and within each of them.

**Table 2:** Synthesis of the epidemiological data.

Countries	Area (km^2^)	Population (2015)	Pop Density / km^2^	# snakebites / year	Incidence /100,000 inhabitants	# deaths /year	Mortality /100.000 inhabitants	CFR (%)	Covered years	Type of data
Argentina	2,766,890	42,669,500	15.42	700	1.64	5	0.012	0.71	2007–2014	Geographic; season; gender^§^; age^§^
Belize	22,966	349,728	15.23	ND	ND	ND	ND	ND	ND	ND
Bolivia	1,098,580	10,027,254	9.13	900	8.98	40	0.399	4.44	1996–2015	Geographic; season; gender; age; altitude^§^
Brazil	8,514,877	203,106,000	23.85	27,200	13.39	115	0.057	0.42	2001–2012	Geographic; season; gender; age
Canada	9,984,670	35,427,524	3.55	100	0.28	0	0.000	0.00	NA	Few envenoming^§^
Chile	756,950	17,773,000	23.48	NA	NA	NA	NA	NA	NA	No front fanged snakes
Colombia	1,138,910	47,757,000	41.93	4,150	8.69	35	0.073	0.84	2009–2014	Geographic; season
Costa Rica	51,100	4,667,096	91.33	700	15.00	7	0.150	1.00	2005–2012	Geographic; season; altitude; gender; age
Ecuador	283,560	15,819,400	55.79	1,500	9.48	9	0.057	0.60	2014–2015	Geographic; season; gender; age
El Salvador	21,041	6,401,240	304.23	300	4.69	ND	ND	ND	2010–2012	Season^§^
French Guiana	86,504	237,549	2.75	50	21.05	1.5	0.631	3.00	1980–1986	§
Guatemala	108,889	15,806,675	145.16	900	5.69	ND	ND	ND	2001–2010	Geographic^§^; age^§^
Guyana	214,999	784,894	3.65	200	25.48	3	0.382	1.50	2010–2012	Geographic^§^; age^§^
Honduras	112,492	8,555,072	76.05	650	7.60	ND	ND	ND	2009–2013	Geographic; season
Martinique	1,128	392,291	347.78	15	3.82	0.2	0.051	1.33	1991–2010	§
Mexico	1,964,375	119,713,203	60.94	4,000	3.34	50	0.042	1.25	1998–2001	Geographic; gender
Nicaragua	130,373	6,071,045	46.57	650	10.71	7	0.115	1.08	2005–2009	Geographic; season; altitude; age
Panama	75,417	3,405,813	45.16	1,900	55.79	15	0.440	0.79	2005–2009	§
Paraguay	406,750	6,783,374	16.68	250	3.69	5	0.074	2.00	2004–2015	Geographic; season
Peru	1,285,220	30,814,175	23.98	2,150	6.98	10	0.032	0.47	2000–2015	Geographic; season
Saint Lucia	539	180,000	0.33	ND	ND	ND	ND	ND	ND	ND
Suriname	163,270	534,189	3.27	ND	ND	ND	ND	ND	ND	ND
Trinidad	4,827	1,267,144	262.51	ND	ND	ND	ND	ND	ND	ND
United States	9,629,091	320,206,000	33.25	5,000	1.56	5	0.002	0.10	1959–2010	Geographic^§^; season^§^; age^§^
Uruguay	176,220	3,286,314	18.65	80	2.43	2	0.061	2.50	1986–2011	Geographical^§^; seasonal^§^
Venezuela	916,445	30,206,307	32.96	5,700	18.87	32	0.106	0.56	1995–2012	Geographic; season; gender; age^§^
**Total**	39,916,083	932,061,967	23.35	57,095	6.13	342	0.037	0.60		-

* = Case fatality rate;

** = Ministry of Health;

^§^ = Bibliography; ND = No data; NA = Not applicable;

^#^ non representative data.

The data are detailed for each country according to the websites mentioned in Table 1, eventually temperate by recent epidemiological or clinical publications.

### Argentina

Information is available online since 2007.

**Fig 1:**
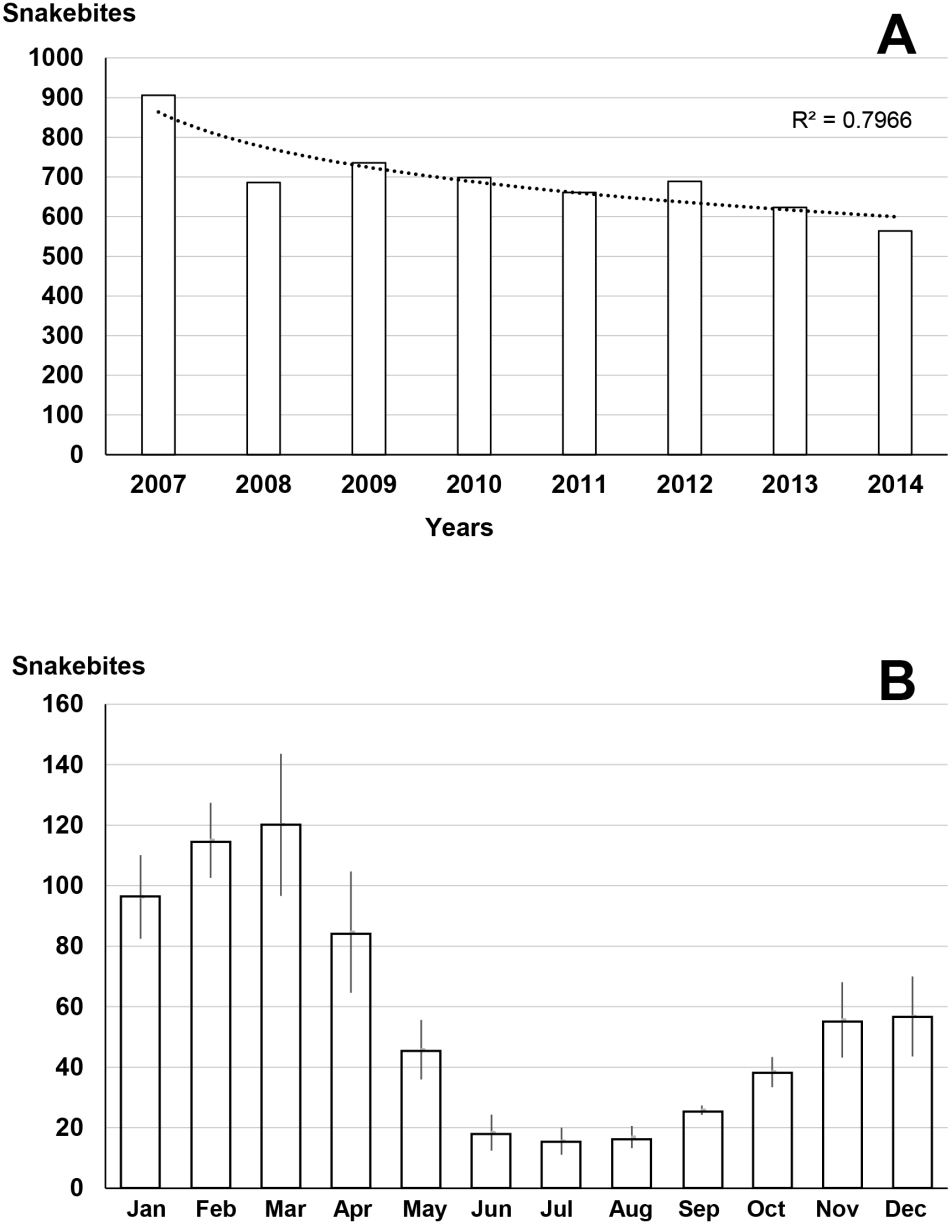
Evolution of snakebites according to time in Argentina. A: annual incidence per 100,000 population from 2007 to 2014. B: average seasonal incidence.

**Fig 2:**
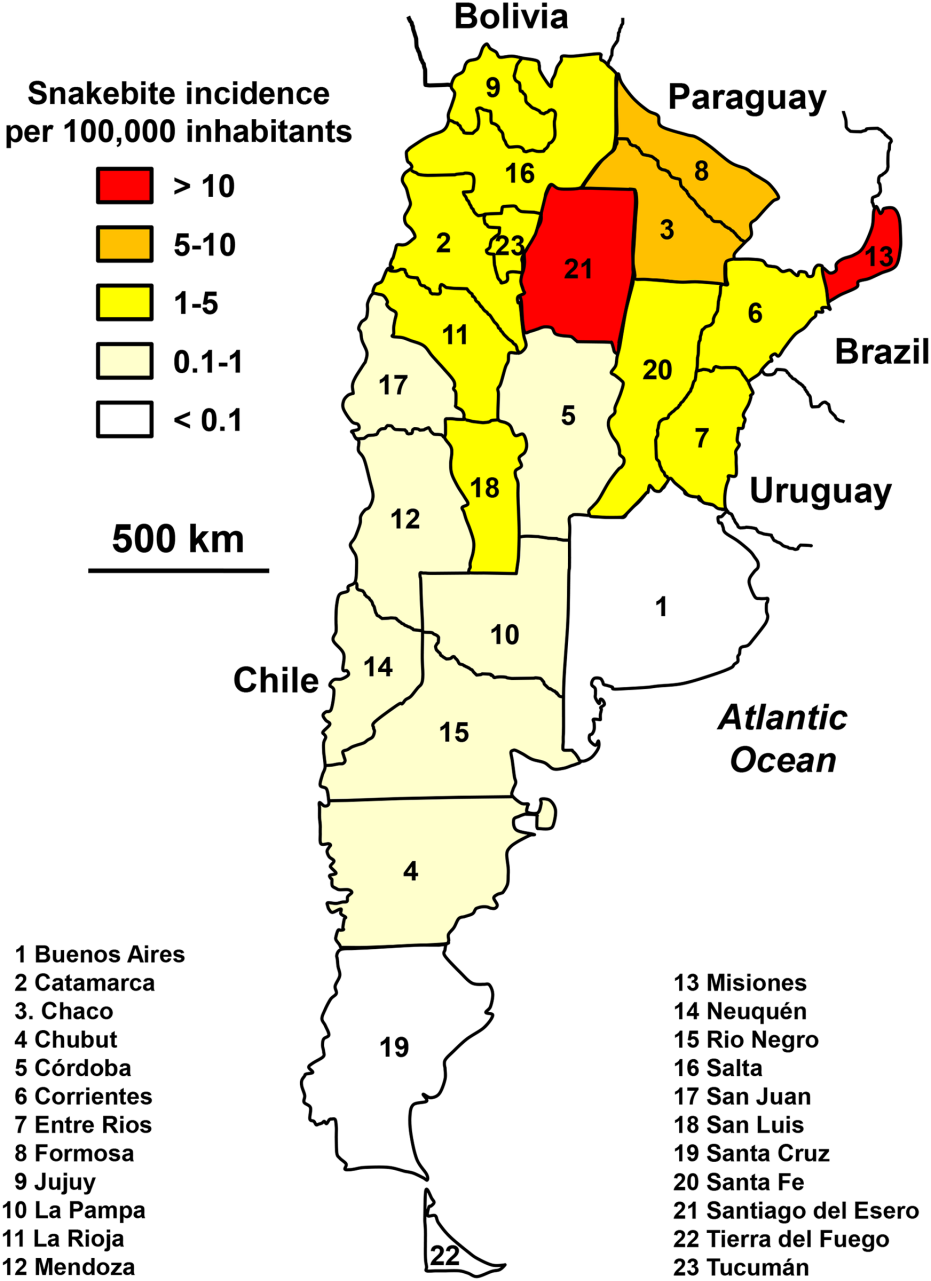
Geographical distribution of incidence of snakebites in Argentina 2007–2014 (Andres Rojas, *Political Map of Argentina*. Available from https://commons.wikimedia.org/wiki/Atlas_of_Argentina#/media/File:Argentina.svg [07/24/2016]).

According to Ministry of Health records, there was an average of 700 envenomations (1.63 per 100,000 inhabitants) and 5 deaths per year (0.012 per 100,000 inhabitants) during the 2007–2014 period. There was a steady decrease in annual incidence (Fig 1A). The incidence showed a decreasing gradient from north to south (Fig 2), which corresponded to the climatic trend between the Chaco province which climate is subtropical, and the Patagonia province more rigorous on the one hand, and the Andean climate of eastern Argentina, on the other hand. Two provinces presented a higher incidence than the others: Santiago del Estero in the north with a low population density (7 inhabitants per km^2^) and Misiones in the north-east with a higher density (35 inhabitants per km^2^) but predominantly agricultural. The seasonal distribution of envenomation showed a summer incidence five to six times higher than the winter one (Fig 1B).

These results corroborated those by Dolab et al. [18] obtained from a questionnaire survey conducted at health facilities. These authors have shown the strong geographical heterogeneity of the incidence which can reach 150 envenimations per 100 000 inhabitants in certain places. They confirmed the low case fatality rate (0.04% according to the survey). *Bothrops* were responsible for 96.6% of the bites, *Crotalus* 2.8% and *Micrurus* 0.6%. Population at risk consisted of young men bitten during agricultural activities. Most envenomations (90%) were treated within the first four hours by an antivenom.

### Belize

No information on snakebites has been obtained for Belize.

However, on the basis of existing data from neighboring countries showing similar environments, the annual number of bites can be estimated at 35 (10 per 100,000 population) and deaths at 1 every 2 to 4 years (0,1 per 100 000 inhabitants).

### Bolivia

The information is available online from 1996 but the notification was interrupted between 2001 and 2009. It returned to availability from 2010. The presentation of the data has been standardized, in particular as regards the classification of age groups. By 2015, the information has been supplemented by the addition of the gender of the patients. However, mortality from envenomation is still not provided.

In their study on snakebites in Bolivia, Chippaux and Postigo [19] reported a national incidence of 8 bites per 100,000 population per year with a case fatality rate of 0.42 per 100,000 population. They extrapolated mortality from household survey data, which lacks precision and reliability but was consistent with the mortality observed in neighboring countries. Updating data available up to 2015 confirmed the impact over this period with over 900 annual bites (9.1 per 100,000 population). The number of deaths is still not reported but has been estimated at around 40 per year [19]. The main results of these authors, in particular the geographical distribution and the distribution by age group, were confirmed by the notifications during the years 2010–2015.

**Fig 3:**
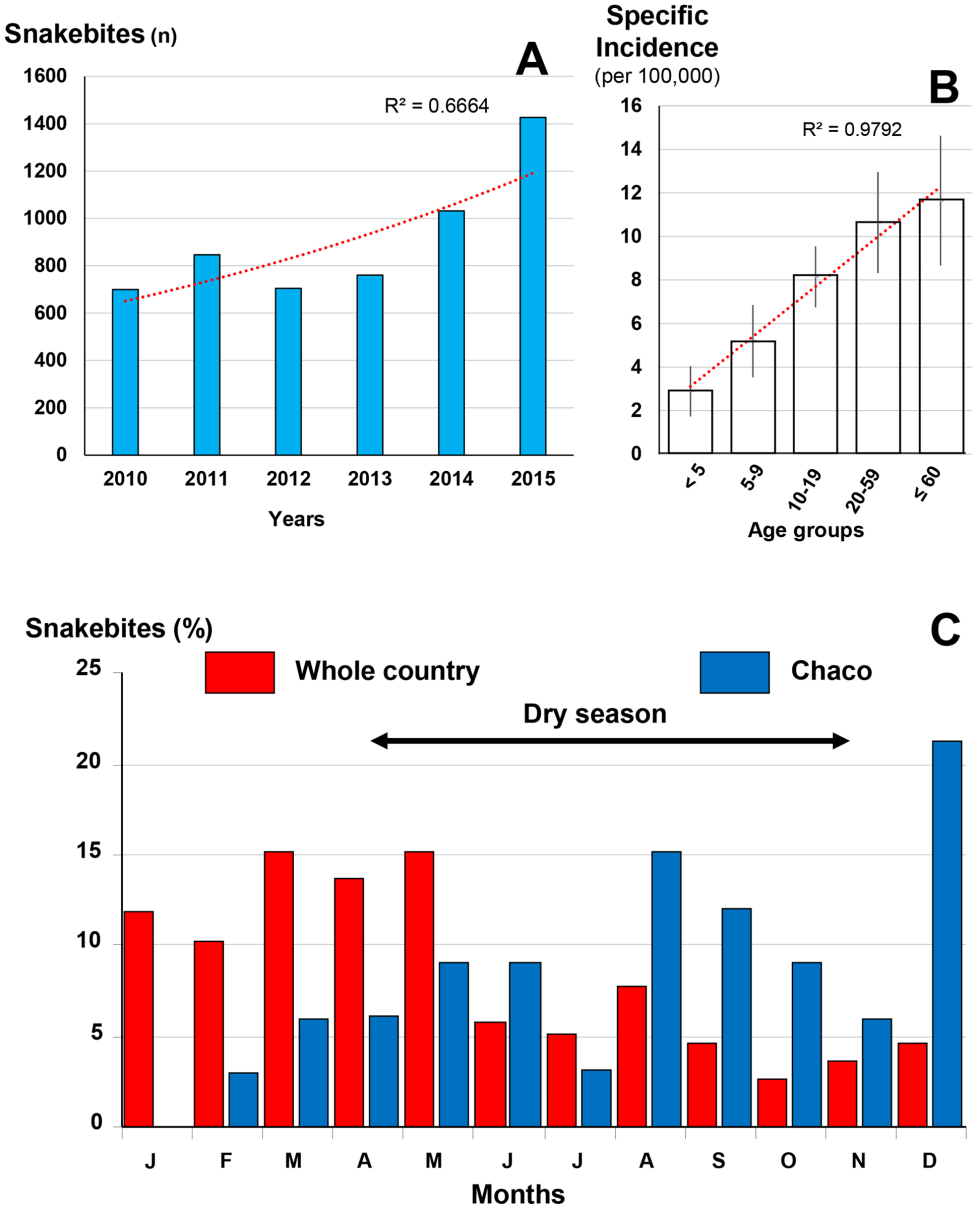
Epidemiological presentation of snakebites in Bolivia. A: annual evolution of bites between 2010 and 2015. B: Incidence of snakebites per 100,000 population of each age group between 2010 and 2015. C: comparison of the seasonal incidence of snakebites between 1996 and 2000.

The annual incidence increased significantly between 2010 and 2015 (Fig 3A). The distribution of the specific incidence showed a steady growth according to age (Fig 3B). The sex ratio (M/F) was 1.81. The geographical distribution was very heterogeneous. The incidence is very low (less than 1 snakebites per 100,000 inhabitants) in the high mountain region, notably the Altiplano (departments of Potossi, Oruro and most of that of La Paz where altitude exceeds 3,500 m asl). The lowland or steppe departments, such as the Chaco region (Departments of Tajira, Santa Cruz, Chuquisaca, Cochabamba and Beni) have an incidence of between 5 and 50 per 100,000 inhabitants. Finally, the incidence exceeds 50 bites per 100,000 inhabitants in the Department of Pando in the Bolivian Amazon [19]. The seasonal distribution (Fig 3C) showed a clear difference between the Chaco province (medium-altitude steppe) where the incidence is highest in the dry season, and Amazonia (low-lying primary forest) where bites occur mainly during the season rains. Finally, if the relationship between population density and incidence was not shown, Chippaux and Postigo [19] observed a significant inverse correlation (P < 1.6·10^−4^) between incidence and altitude.

### Brazil

The notification of snakebites is performed for a long time in Brazil but the results are online only since 2001. According to Chippaux [20] from the data reported by the health facilities and available online on the site of SINAN which is the main Database on causes of morbidity and mortality available online since 2001 [21], the average number of snakebites was about 27,200 per year (15 per 100,000 population) with more than 115 deaths (0.06 100,000 inhabitants) during the period 2001–2012.

**Fig 4:**
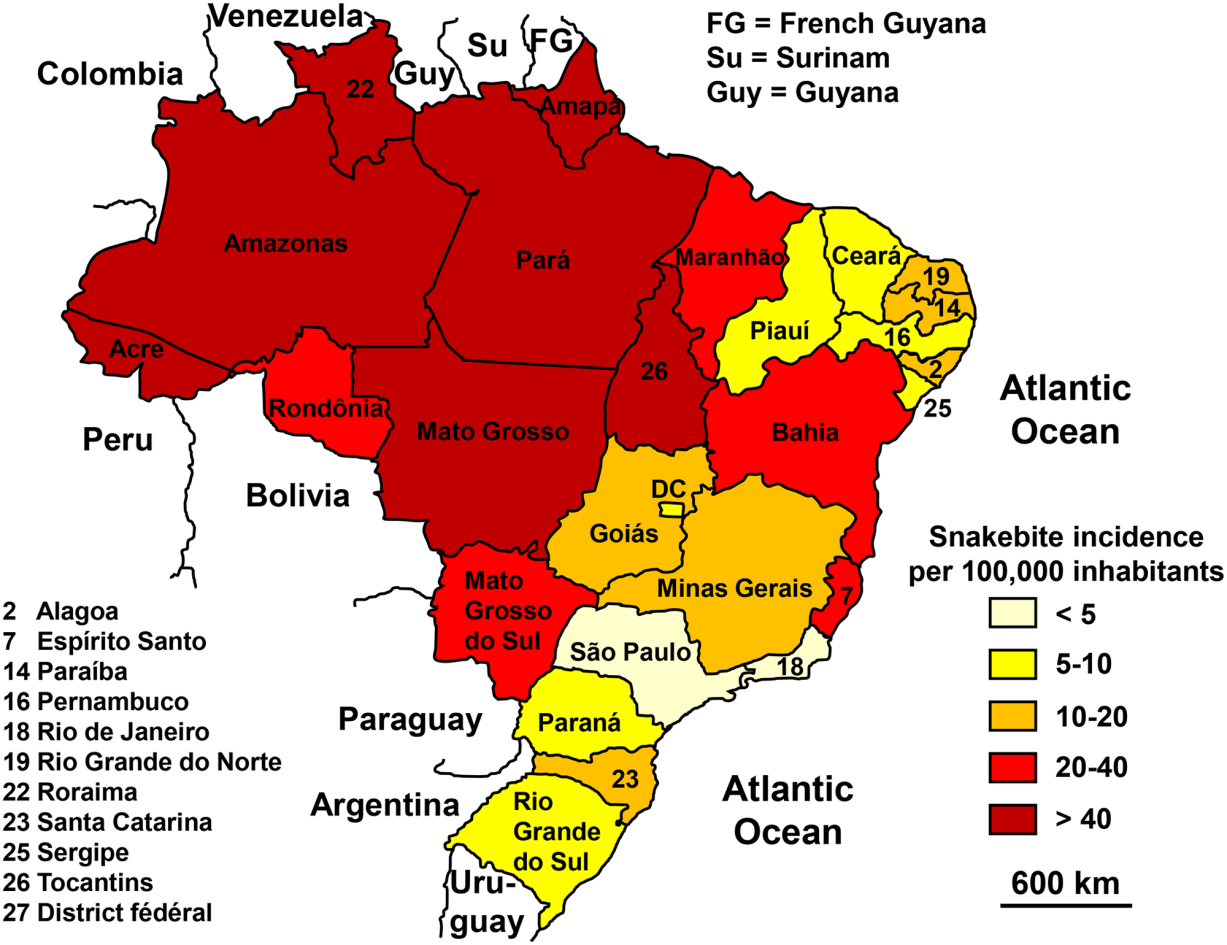
Geographical distribution of incidence of snakebites in Brazil 2007–2012 (Raphael Lorenzeto de Abreu, *Map of Brezil’s region*. Available from https://commons.wikimedia.org/wiki/Atlas_of_Brazil#/media/File:Brazil_Regions.svg [07/24/2016]).

**Fig 5:**
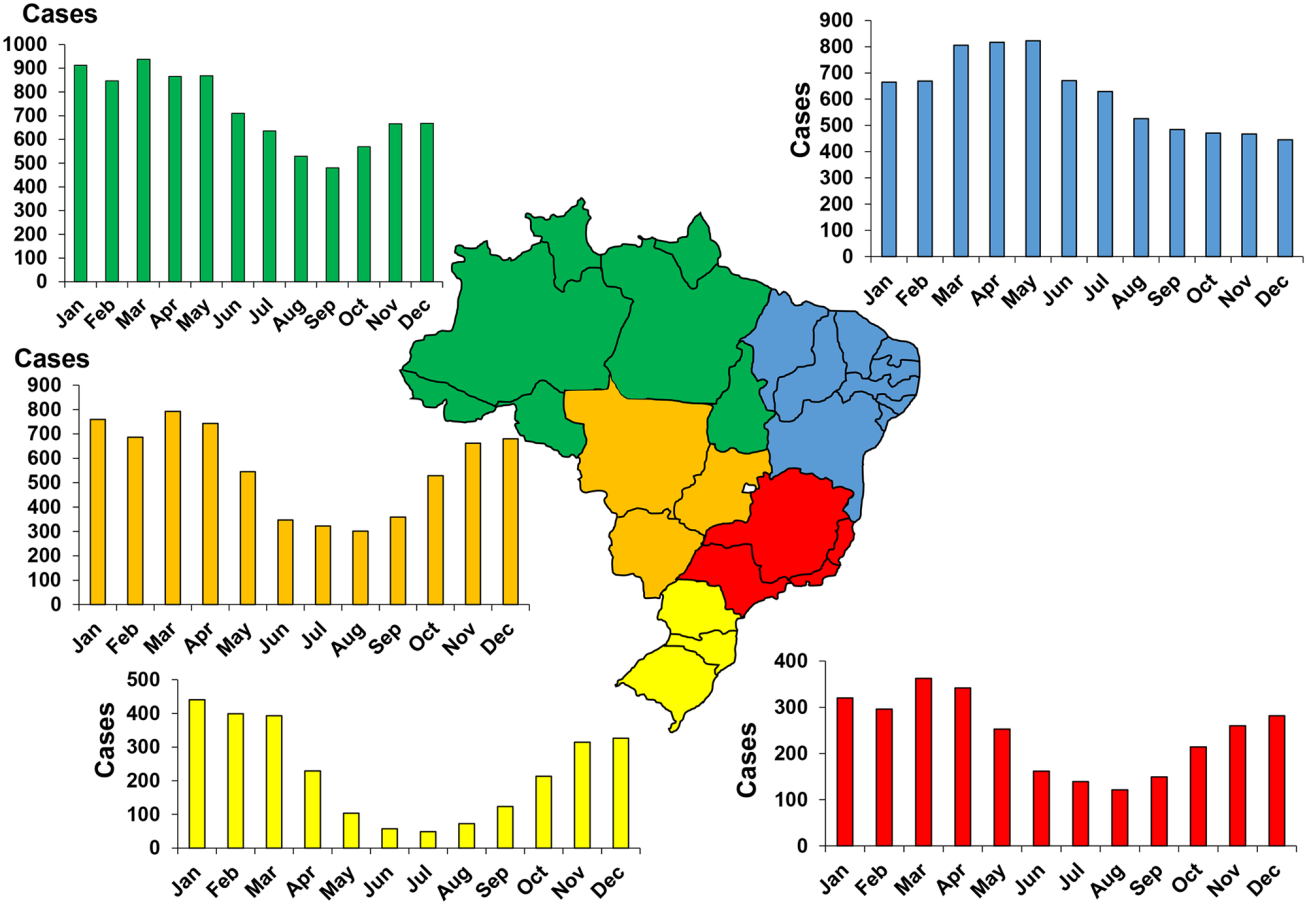
Regional variation in the seasonal incidence of snake bites in Brazil between 2007 and 2012.

The geographical distribution showed a clear predominance in northern Brazil, especially in the Amazon (Fig 4). The seasonal distribution of bites was more pronounced in the summer, particularly in the southern regions (Fig 5). The incidence by age group varied greatly from region to region. It was higher among young people in the Amazon and in people over the age of 40 on the inland plateau [20]. *Bothrops* species were responsible for most of the bites everywhere in Brazil. Bites by *Crotalus durissus* are more frequent in the eastern and central savannas. The bites by *Lachesis* sp. are mostly observed in the Amazonian region. Those by *Micrurus* sp. are rare. Finally, there was a strong inverse correlation between incidence and population density [20].

The population at risk was made up of male farmers. Risk factors were more or less directly related to the agriculture and rural housing of the victims [22; 23]. Bochner and Struchiner [24] showed that these characteristics have been constant since the first epidemiological studies carried out by Vital Brazil in the early 20^th^ century.

Incidence and mortality increased discreetly and seemed to follow demographic trends [20].

### Canada

Snakebites appeared to be very rare in Canada because of a climate unfavorable to the establishment of snake populations, and a highly mechanized agricultural activity. The presence of *Sistrurus catenatus* is attested in southern Ontario, the most populated region of the State, and *Crotalus oreganus* occurs in British Columbia. *Crotalus horridus* disappeared from Oregon since 1941 and from Quebec more recently [25]. Rumors of his return to southeastern Canada, including Quebec, have not been validated by the Recovery Commission for the Ontario Rattlesnake [26].

According to Dubinsky [27], there were about sixty snakebites reported in Ontario each year. There would have been 2 deaths between 1900 and 1960 [28] and since the 60s none has been reported in the literature. There were no figures for British Columbia and snakebites are considered very rare.

In total, it can be assumed that snakebites are fewer than 100 annually and no death was reported in Canada since a long time. Snakebites are distributed in the two southern states (Ontario and British Columbia) close to the border of United States of America where rattlesnakes are still encountered. However, some snakebites recorded could be illegitimate bites inflicted when manipulating a snake in the field or in captivity.

### Chile

There are neither *Bothrops*, nor *Crotalus*, nor *Micrurus* in Chile. Snakebites by opistoglyphic snakes were reported but not considered as public health issue [29].

### Colombia

Notifications have been available online since 2009. The number of reported snakebites was approximately 4,150 per year (8.5 per 100,000 population), resulting in about 35 deaths (0.08 per 100,000 population) between 2009 and 2014.

**Fig 6:**
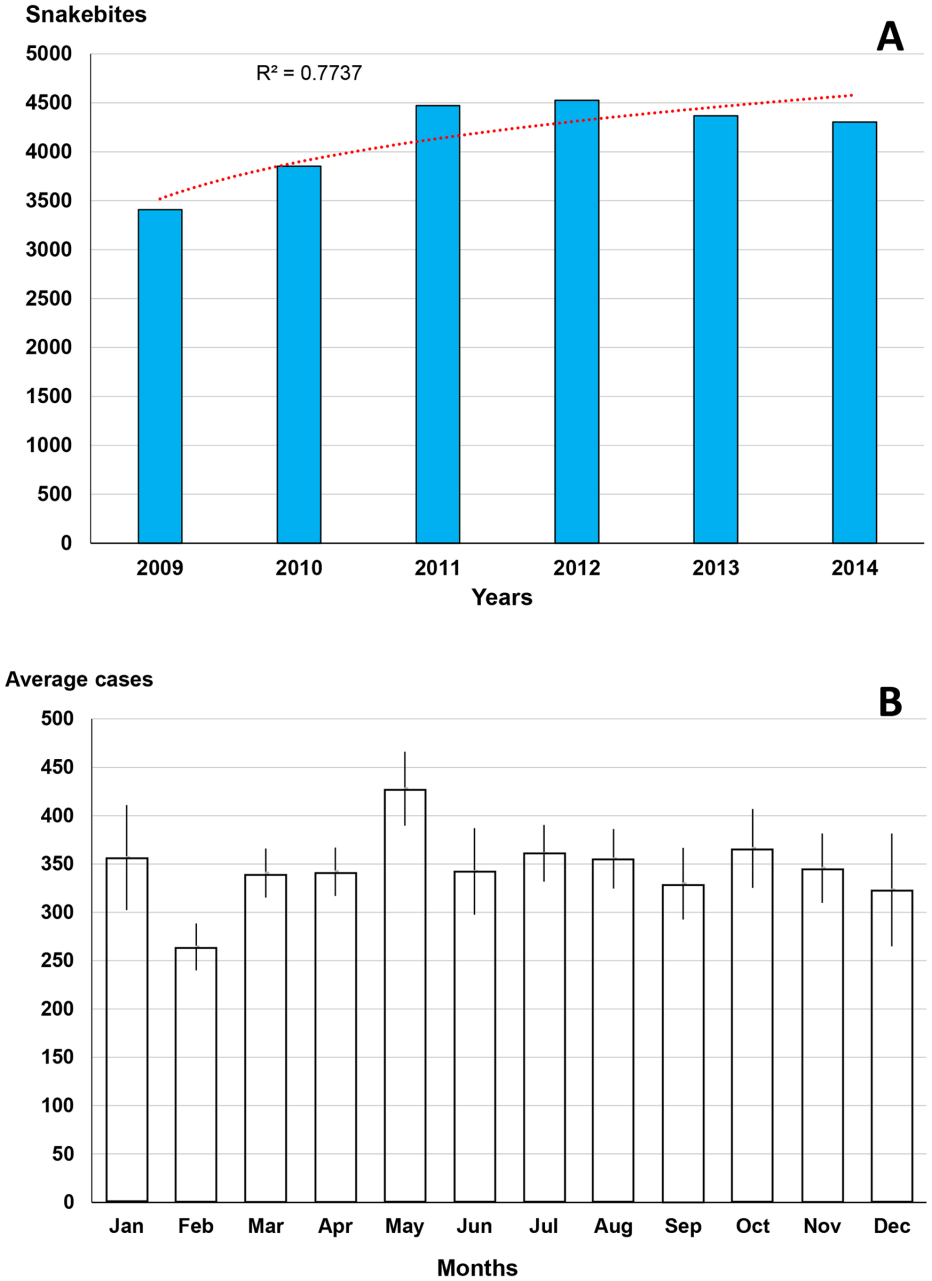
Evolution of snakebites according to time in Colombia. A: annual incidence from 2009 to 2014. B: average seasonal incidence (2009–2014).

**Fig 7:**
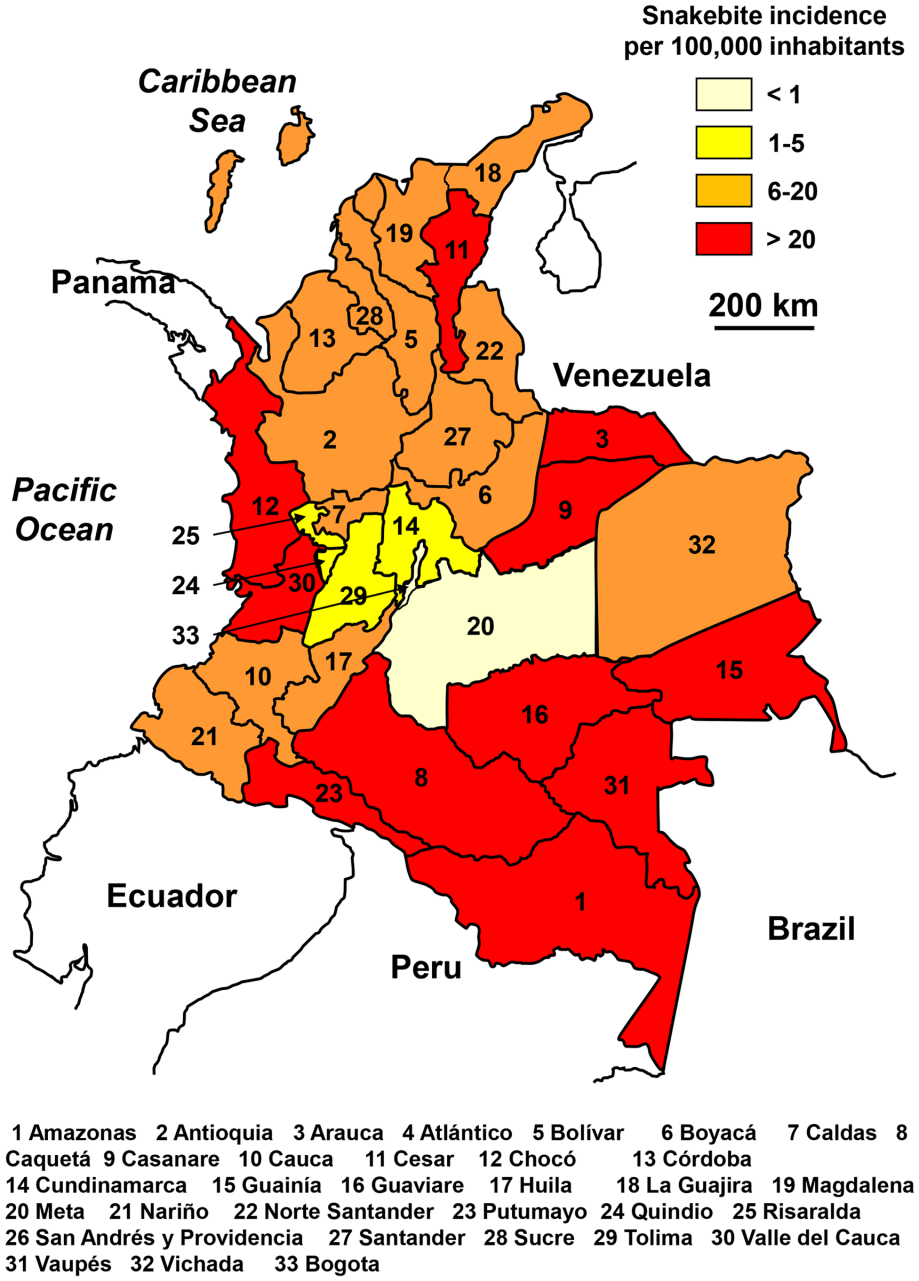
Geographical distribution of incidence of snakebites in Colombia 2009–2014 (canislupusarctos, *Colombia and the departementos*. Available from https://commons.wikimedia.org/wiki/Atlas_of_Colombia#/media/File:Colombia_departaments-numbered.svg [08/23/2015]).

The incidence of snakebites increased significantly during the period (Fig 6A) without clear explanation. Maybe, the case report system–or political situation–improved enough to obtain more reliable data. The geographical distribution was heterogeneous. Incidence was relatively high in the whole of the country, especially in the Amazonian departments in the south (Fig 7) and much lower in central Colombia, both mountainous and urban. There was no correlation between population density and incidence. The seasonal distribution was constant throughout the year (Fig 6B).

### Costa Rica

The notification has been available online since 2005 with some shortcomings—or delays in data capture—after 2012.

From 2005 to 2012, an average of nearly 700 snakebites (15 per 100,000 inhabitants) and 7 deaths (0.15 per 100,000 inhabitants) were reported.

**Fig 8:**
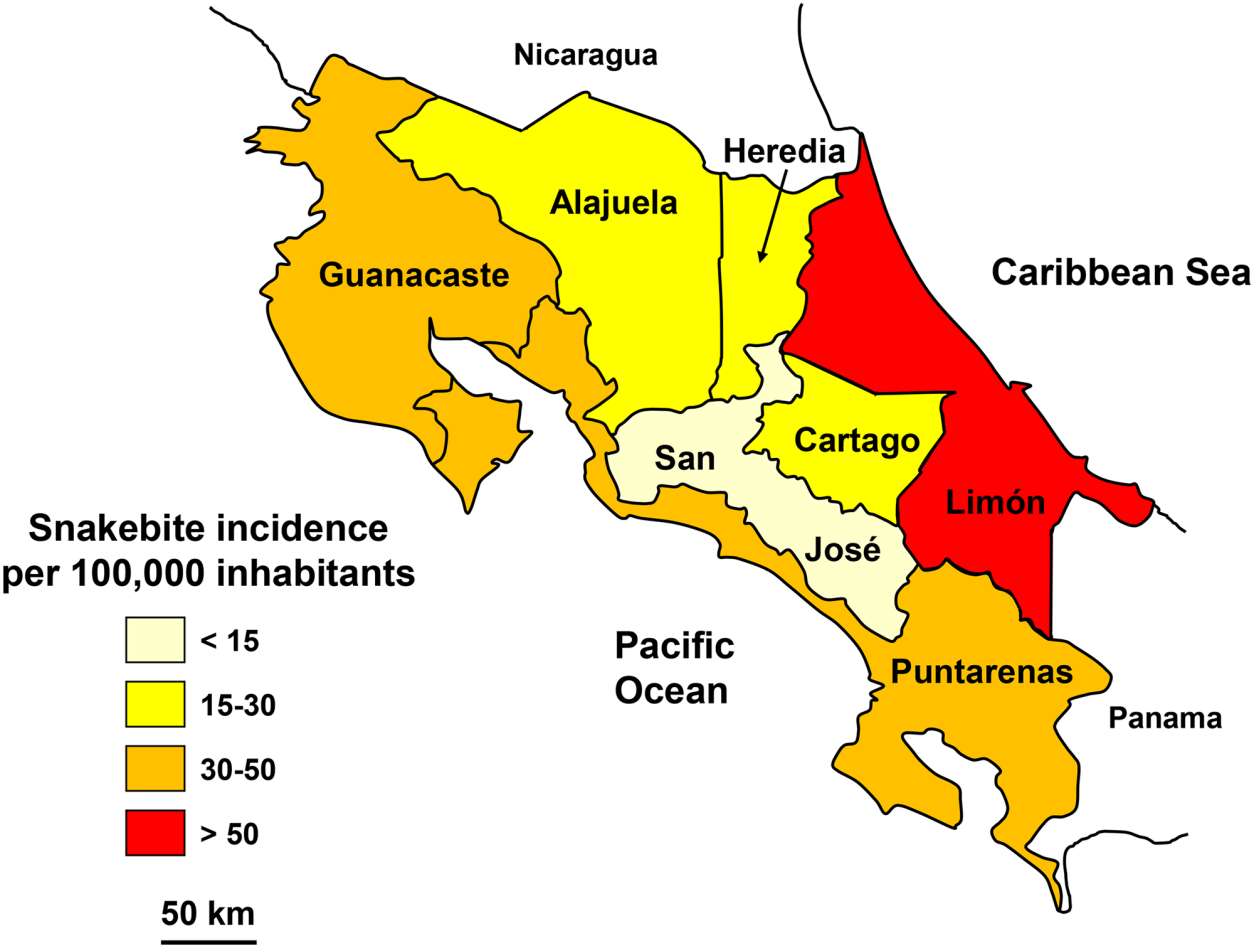
Geographical distribution of incidence of snakebites in Costa Rica 2005–2012 (Golbez, *Map of the Provinces of Costa Rica*. Available from https://commons.wikimedia.org/wiki/Atlas_of_Costa_Rica#/media/File:Costa_Rica_provinces_named.png [08/24/2015]).

**Fig 9:**
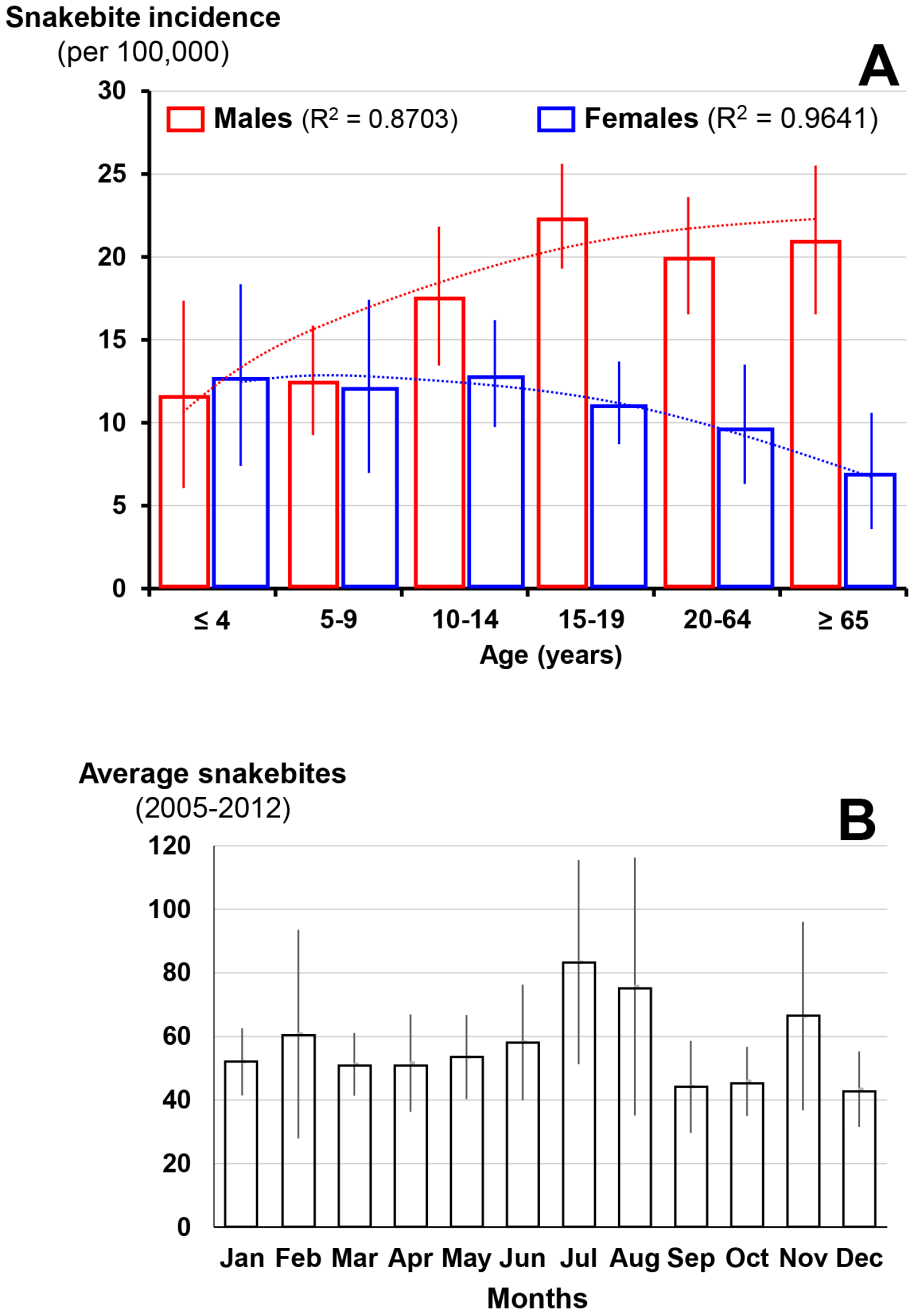
Epidemiology of snakebites in Costa Rica. A: Age and gender specific incidence (per 100,000 population of each age and gender groups) of snakebites in Costa Rica 2005–2012. B: Seasonal incidence of snakebites in Costa Rica 2005–2012.

The incidence of snakebites was significantly higher in the eastern provinces. In the center of the country, it was lower, especially in the province of San José, which is the most densely populated and mountainous (Fig 8). Annual snakebites ranged 500–1,000 without any particular trend [30]. The sex ratio was 1.7 (M/F) and increased in adult male whereas it decreased in women over 15 years (Fig 9A). The seasonal incidence was relatively stable during the year, however, with marked variability in the rainy season from May to November when the majority of snakebites occurred (Fig 9B).

These results were in agreement with those from the literature. The highest mortality is observed in the provinces of Puntarenas in the south and Limon in the east, linked to the abundance of *Bothrops asper* [31; 32]. Based on the notification of cases and environmental information, Hansson et al. [33] were able to model high risk zones of bites by *Bothrops asper*, and to recommend a targeted supply of antivenoms.

### Ecuador

Access to full epidemiological data for years prior to 2013 was limited [34]. From 2013, the weekly notification was available online but showed many shortcomings.

According to González-Andrade and Chippaux [34], there were nearly 1,500 snakebites (9.8 per 100,000 inhabitants) resulting in about 10 deaths (0.06 per 100,000 inhabitants) each year.

**Fig 10:**
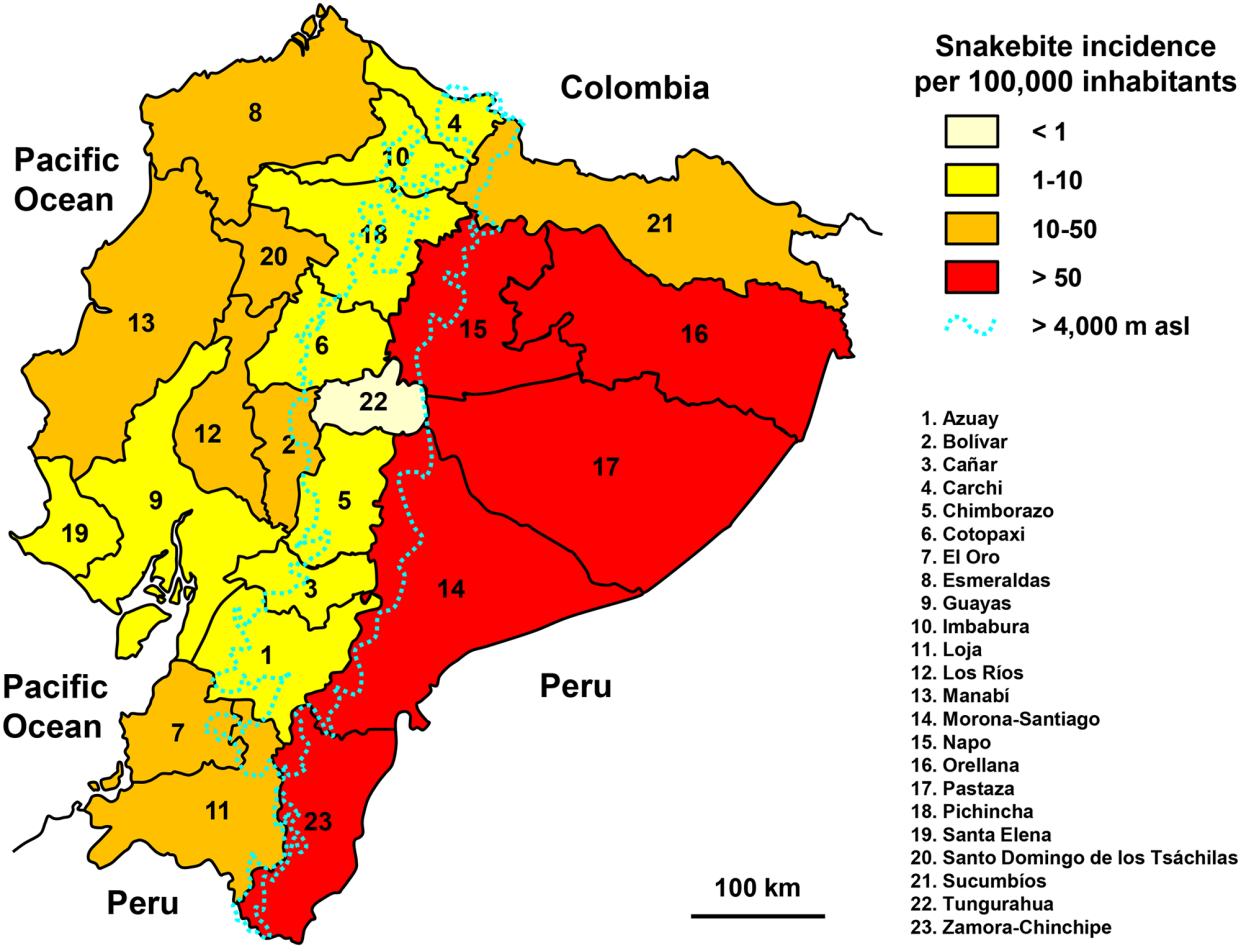
Geographical distribution of incidence of snakebites in Ecuador 2001–2007 (Anonymous, *Provinces of Ecuador*. Available from https://commons.wikimedia.org/wiki/Atlas_of_Ecuador#/media/File:Provinces_of_ecuador.png [08/23/2016] and Anonymous, *Map of Ecuador Demis*. Available from https://commons.wikimedia.org/wiki/Atlas_of_Ecuador#/media/File:Map_of_Ecuador_Demis.png) [08/23/2016].

The highest incidence occurred in the Amazonian provinces (Oriente province), with 37% of the envenomations and an average annual incidence of 100 envenomations per 100,000 inhabitants (Fig 10). The majority of snakebites (58%) were in the coastal region (Costa province) with an average incidence of 12 bites per 100,000 inhabitants. In highland provinces in the center of the country (Sierra province), incidence was about 5 bites per 100,000 population (5% of the snakebites). During the rainy season, from January to April, the incidence of snake bites is twice as high as in the dry season. The incidence of snakebites is twice as high after the age of 10 and remains stable from teenagers to elderly. The very young children below 5 are ten times less involved than adults.

### El Salvador

Although notification of snakebites has been mandatory since 2010, data are not accessible. On the other hand, they are subject to periodic reports put online. We used that of 2013 which compiled the data from 2010 to 2012.

**Fig 11:**
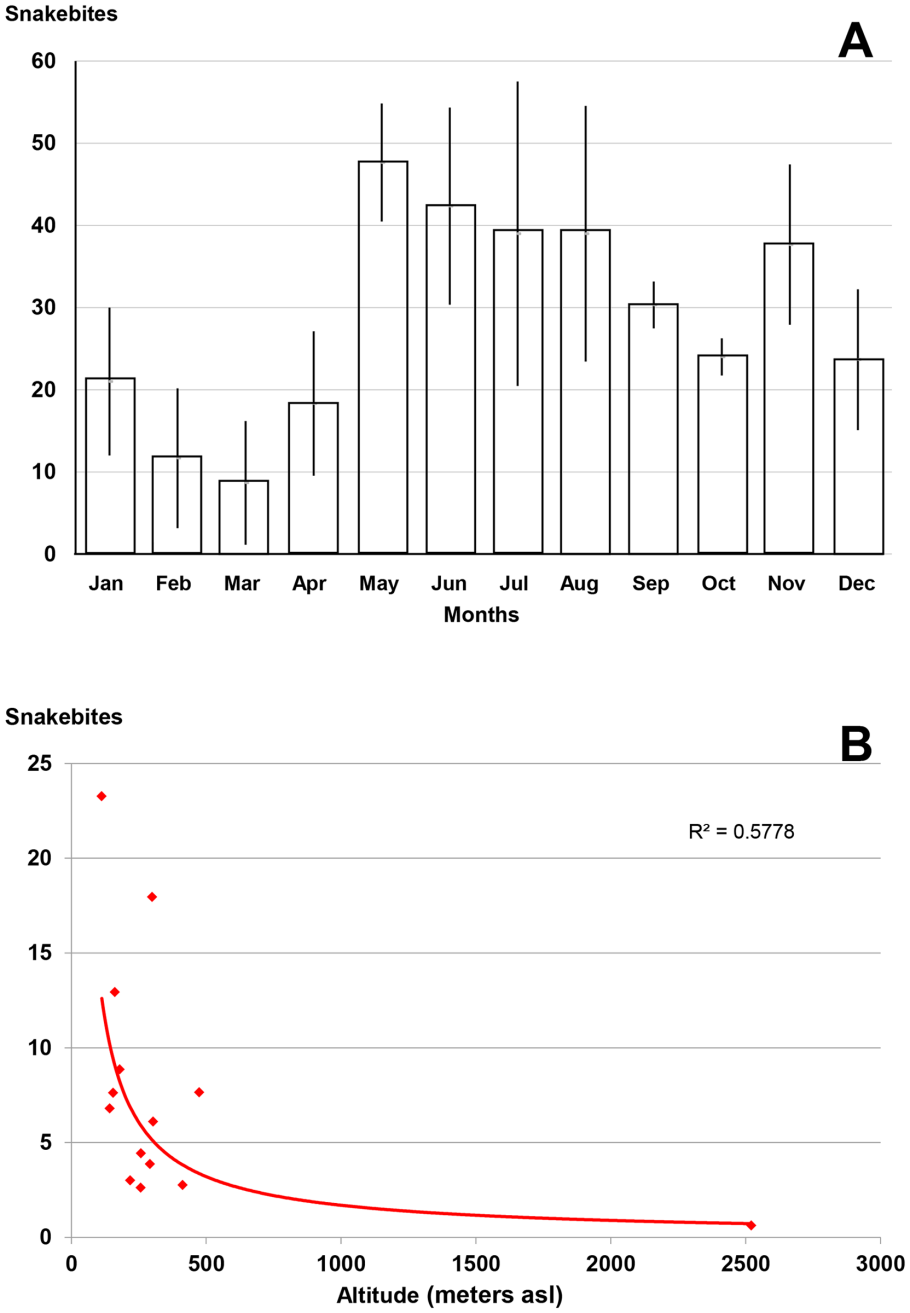
Epidemiology of snakebites in El Salvador. A: Seasonal incidence of snakebites in El Salvador 2010–2012. B: Correlation between altitude and annual incidence of snakebites (per 100,000 population) in El Salvador.

**Fig 12:**
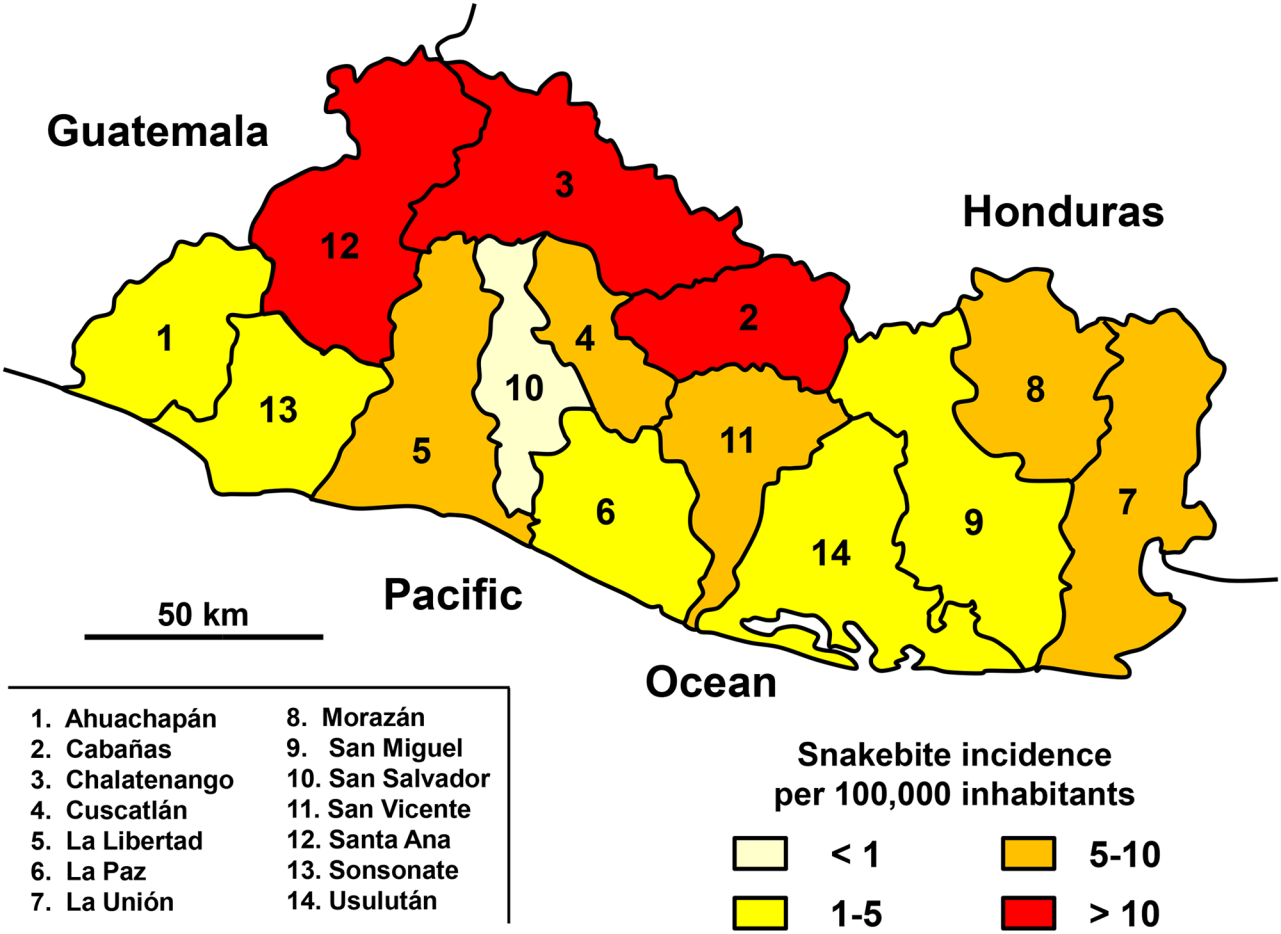
Geographical distribution of incidence of snakebites in El Salvador 2010–2012 (Anonymous, *Departments of El Salvador*. Available from https://commons.wikimedia.org/wiki/Atlas_of_El_Salvador#/media/File:El_Salvador_departments_named.png [08/27/2015].

About 300 annual snakebites (5 per 100,000 inhabitants) were irregularly distributed during the year. Based on the results of neighboring states, the annual number of deaths can be estimated at 3 (0.05 per 100,000 inhabitants). The six months of the rainy season (May to October) accounted for nearly 65% of the envenomations (Fig 11A). The population at risk was mainly composed of young men. Patients aged 10 to 30 constituted 51% of the bites, while this age group represented less than 40% of the population. In addition, the sex ratio (M/F) was 1.5. During this period, no death was reported. The geographic distribution of incidence was heterogeneous, i.e. lower on the coast and in the center of the country (Fig 12), a probable consequence of the local population density, which is the highest of the Americas (Fig 11B).

### French Guyana

There was no recent data concerning this small French department. According to the literature, mostly from surveys dating back to the 1980s, the annual incidence of envenomation exceeded 25 cases per 100,000 inhabitants with relatively high mortality [35–37].

### Guatemala

Data were available online since 2001 with some gaps, notably in 2005.

**Fig 13:**
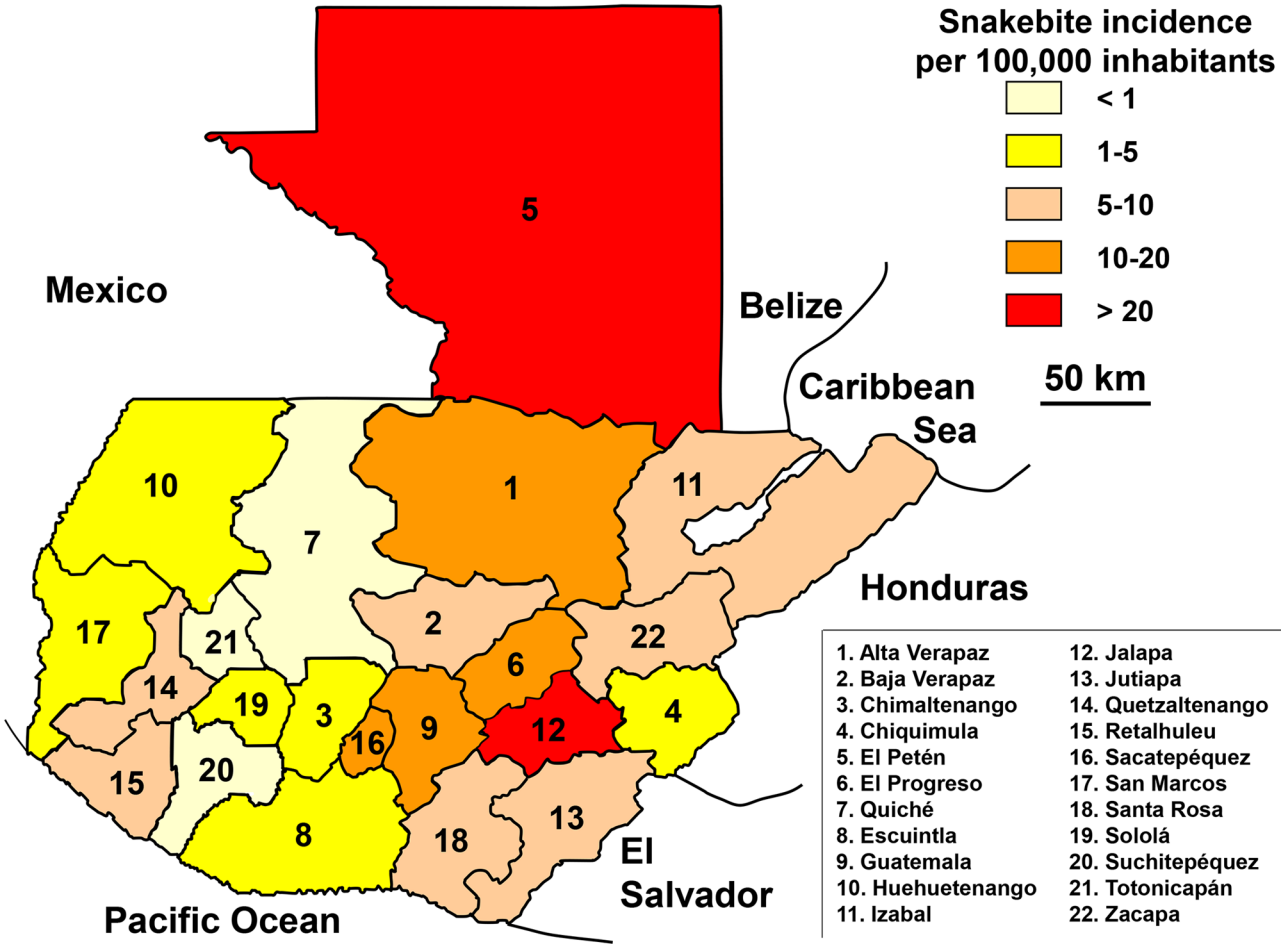
Geographical distribution of incidence of snakebites in Guatemala 2001–2010 (Anonymous, *GuatemalaProvs*. Available from https://commons.wikimedia.org/wiki/Atlas_of_Guatemala#/media/File:GuatemalaProvs.PNG [07/24/2016].

With almost 900 snakebites on average each year (2001–2010), the distribution of the incidence was very heterogeneous (Fig 13). Mortality was not documented. It was estimated on the basis of neighboring country mortality at about 10 deaths per year (0.06 per 100,000 population).

### Guyana

There was no notification of snakebites in Guyana. However, a study of cases of envenomation treated at the Georgetown Public Hospital Corporation (GPHC) in 2014 provided an estimate of the burden of envenomation for Guyana as a whole. However, data for the Amazon region, which is sparsely populated but with high snakebite risk, was highly under-estimated, partly because it was likely that few patients visit the health facilities and, on the other hand because the evacuation possibilities on Georgetown are almost nonexistent.

According to Bux [38], there would be more than 200 snakebites each year in Guyana, an incidence greater than 25 bites per 100,000 inhabitants. The number of deaths was not specified, but Langston [39] mentioned a high number of deaths. The press reported 3 deaths in Georgetown between 2011 and 2014, which was probably underestimated since it did not take into account deaths in provincial health facilities.

**Fig 14:**
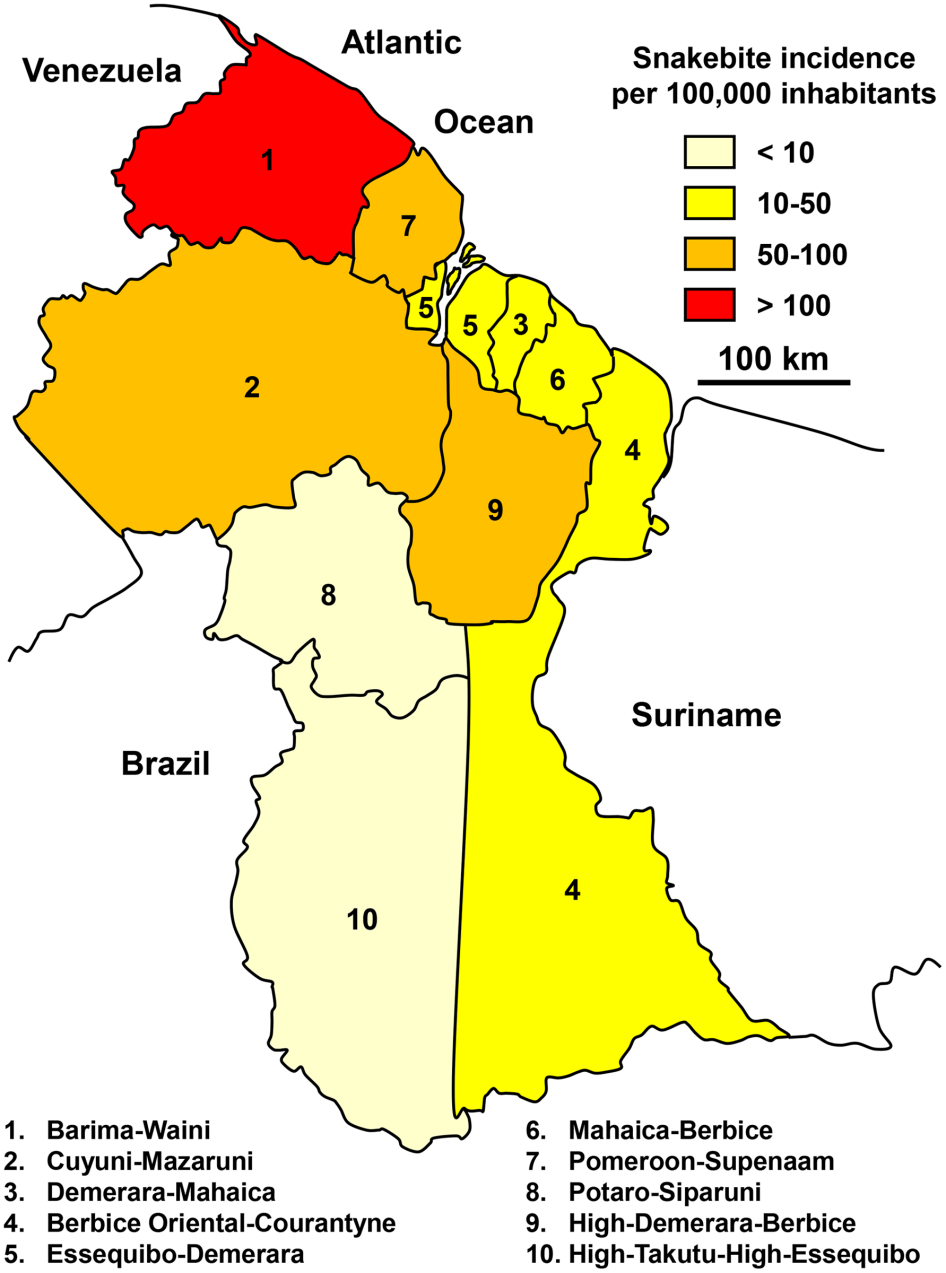
Geographical distribution of incidence of snakebites in Guyana 2010–2012 (Golbez, *Map of the regions of Guyana*. Available from https://commons.wikimedia.org/wiki/Atlas_of_Guyana#/media/File:Guyana_regions_english.png [13/11/2016].

More than 80 snakebites were treated each year at the Georgetown Reference Hospital during the 2010–2012 period. However, the geographical distribution was biased due to the lack of reliable data for the South (Amazonian region) of the country (Fig 14). The age-specific incidence calculated on the basis of hospital data showed a constant increase of snakebite incidence until the age of 30–40 years and then a steady decline up to 60 years.

### Honduras

Notification of snakebites has been mandatory since 2009 but online display was interrupted at the end of 2013.

A little more than 650 snakebites occurred annually on average (10 per 100,000 inhabitants). The number of deaths was not reported but was estimated at 7 per year (0.08 per 100,000 population) based on observations in neighboring countries.

**Fig 15:**
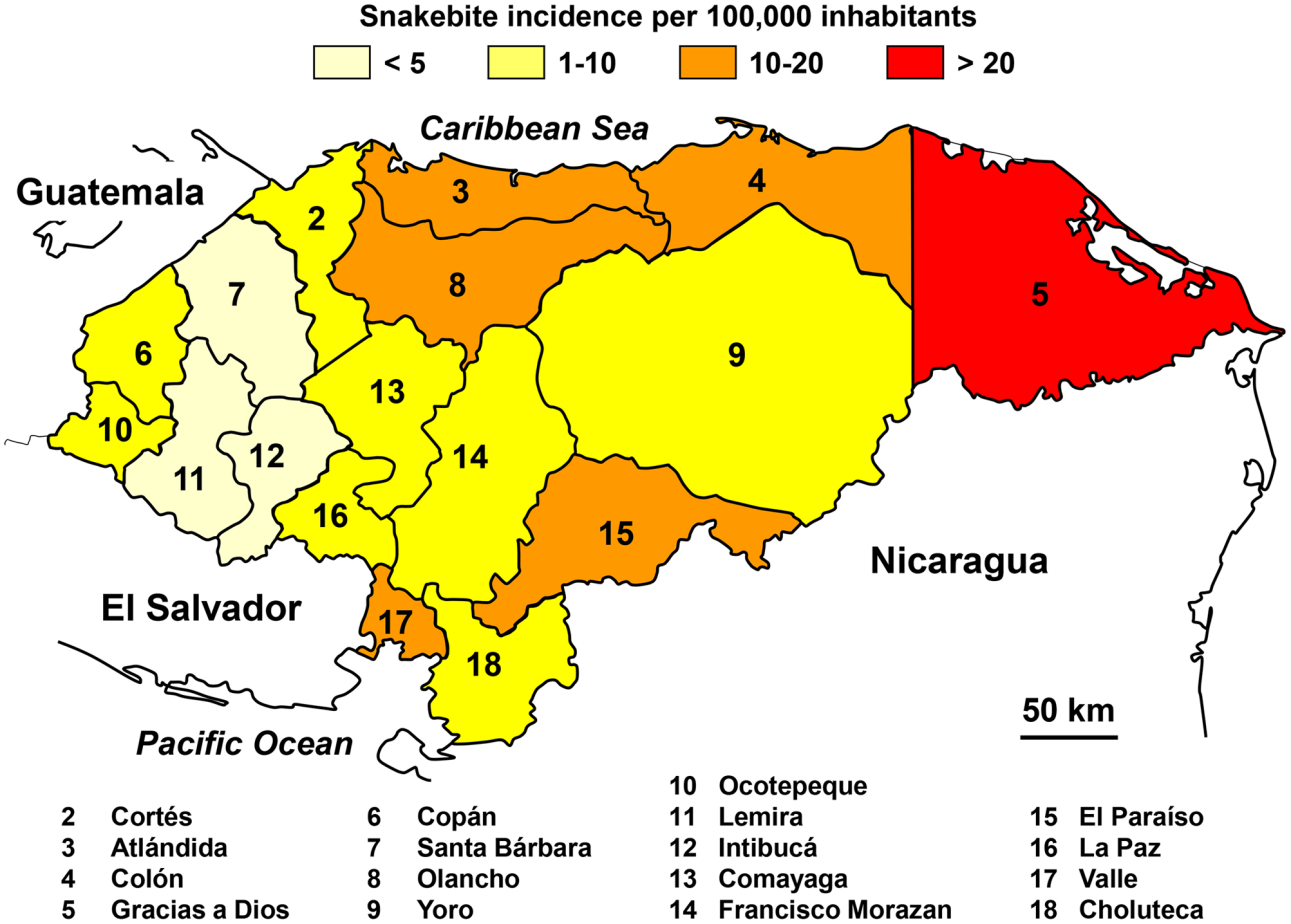
Geographical distribution of incidence of snakebites in Honduras 2009–2012 (Golbez, *Map of the departments of Honduras*. Available from https://commons.wikimedia.org/wiki/Atlas_of_Honduras#/media/File:Honduras_departments_named.png [28/07/2015].

Snakebites were mostly distributed to the north and east of the country (Fig 15), regions with the lowest altitude. The number of snakebites is relatively stable throughout the year with a slight increase in incidence during the rainy season from May to October.

### Martinique

Notification of snakebites was not mandatory in Martinique and records were not available online.

**Fig 16:**
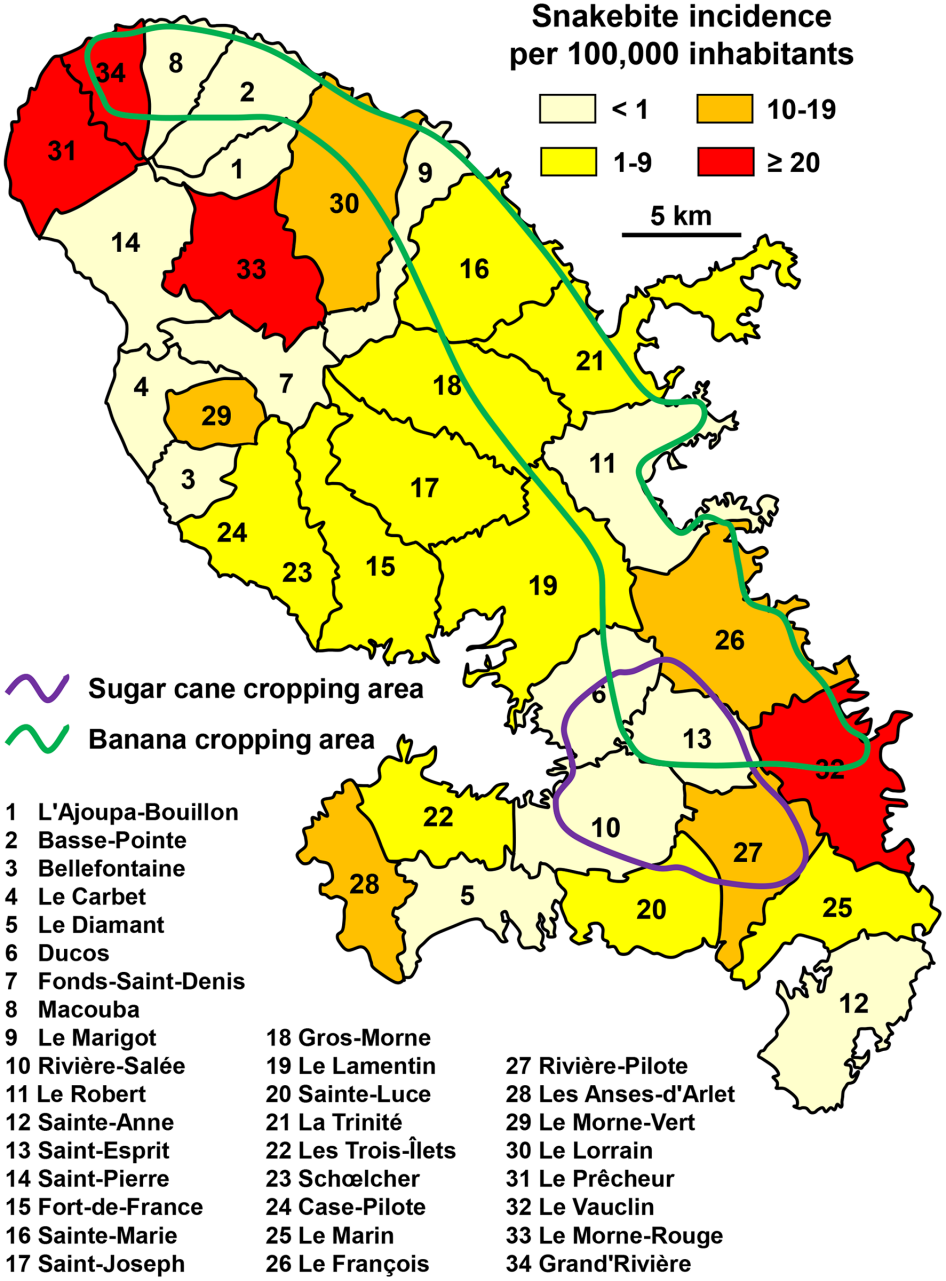
Geographical distribution of incidence of snakebites in Martinique Island 1991–1994 (data from [40]) (BigonL, *Carte de la Martinique avec ses 4 arrondissements*. Available from https://en.wikipedia.org/wiki/Martinique#/media/File:Martinique_legende_arrs.PNG [12/06/2016].

According to the literature [40;41;42], about 15 envenomations are treated in hospital every year (about 5 per 100,000 inhabitants). The average number of deaths was 4 every 20 years (0.05 per 100,000 population) during the 1990–2010 period. Only one venomous species (*Bothrops lanceolatus*) is present on the island [8]. The geographical distribution of the bites covered the whole of the island, but mainly involved small agricultural communes (Fig 16). However, no obvious link was observed between snakebite incidence and agricultural work in the two main types of plantations of Martinique (bananas and sugarcane).

### Mexico

Venomous animal attacks was reported since 1996 but snakebites were separated and available online only since 2003.

**Fig 17:**
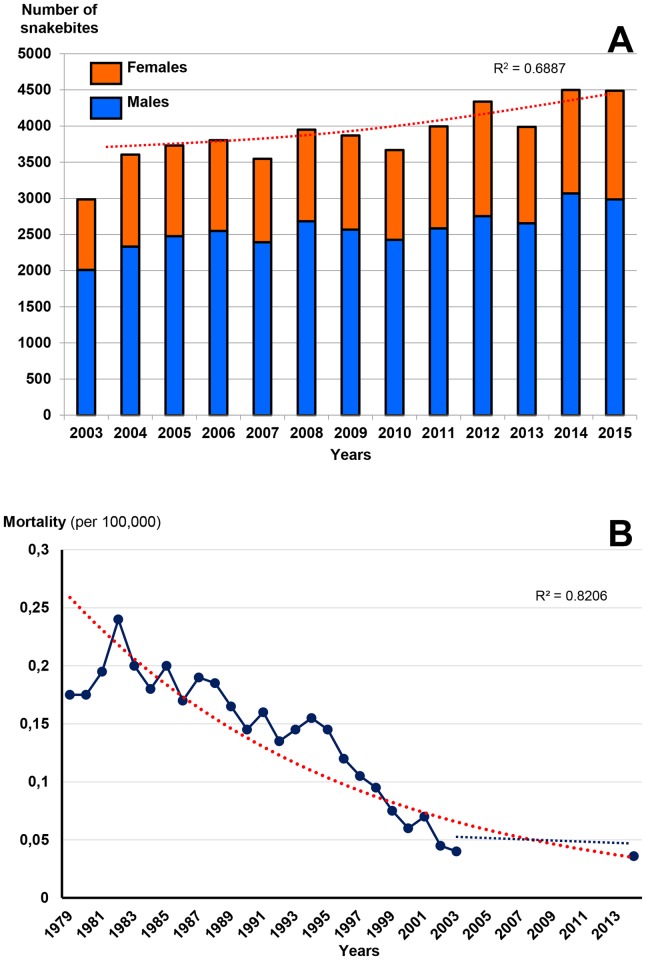
Evolution of snakebites according to time in Mexico. A: annual incidence from 2003 to 2015. B: annual mortality from snakebites in Mexico (1979–2014) [after 43].

**Fig 18:**
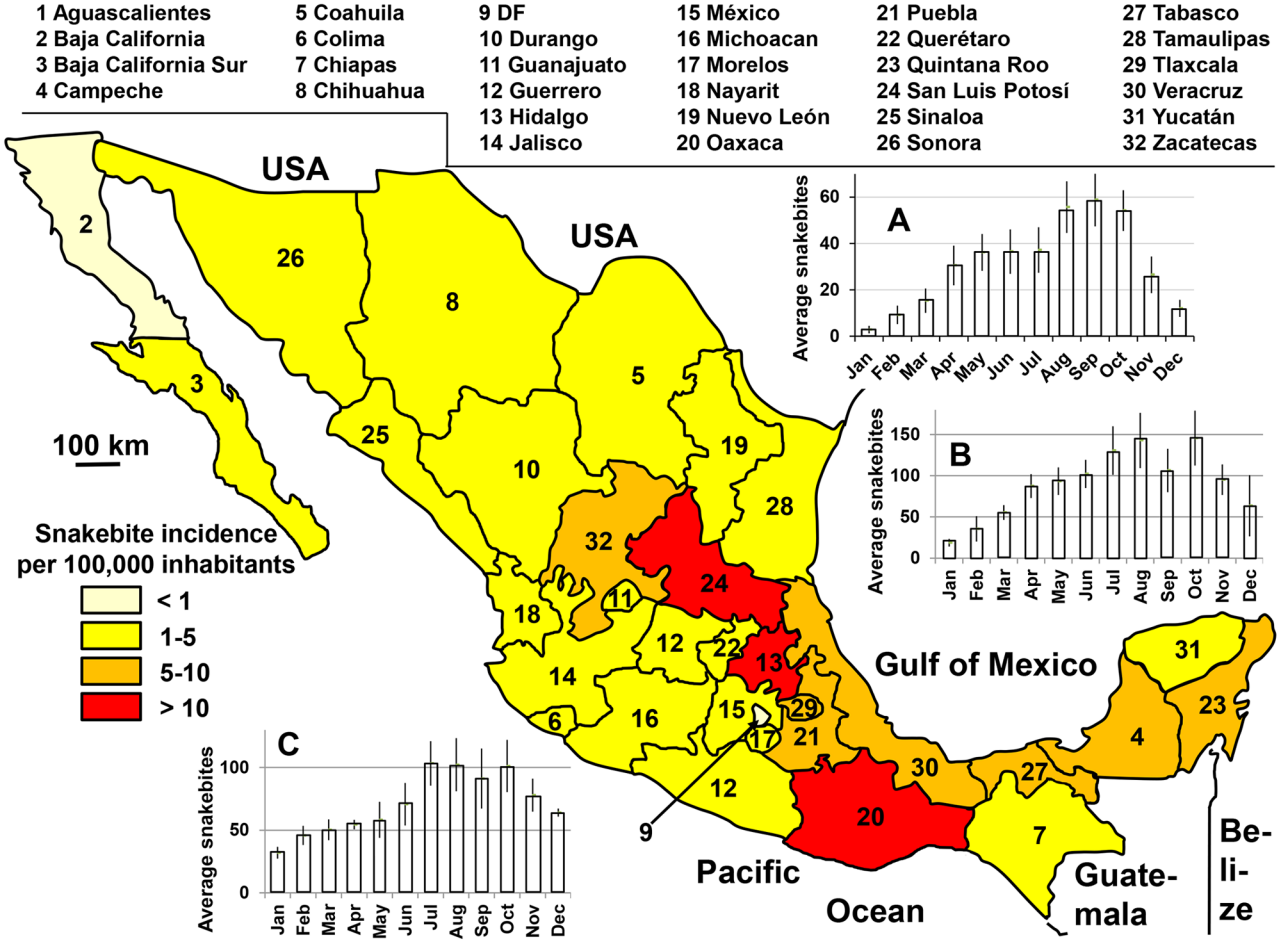
Geographical distribution and seasonal incidence of snakebites in Mexico 2003–2015. A: monthly mean of cases in Northern Mexico (Chihuahua, Cohuila, Nuevo Léon, Sonora, Tamaulipas); B: monthly mean of cases in Central Mexico (Hidalgo, Michoacan, San Luis de Potosi, Puebla); C: monthly mean of cases in Southern Mexico (Campeche, Chiapas, Oaxaca); (Anonymous, *States of Mexico*. Available from https://commons.wikimedia.org/wiki/Atlas_of_Mexico#/media/File:States_of_Mexico.svg [07/10/2016].

The annual number of bites averaged 4,000 (3.3 per 100,000 inhabitants) with steady growth between 2003 and 2015 (Fig 17A). The number of deaths was below fifty per year (0.035 per 100,000 inhabitants). As showed by Frayre-Torres et al. [43], the mortality rate decreased from 0.25 per 100,000 population in the 1970s to 0.05 during the 2000s. The lowering continued after the 2010 and is now less than 0.04 per 100 000 (Fig 17B).In addition, mortality was higher in the South than in the North of Mexico and increased significantly after the age of 40, whereas it appeared to be stable before. Case fatality rate was higher among males than females (P <0.028). The geographical distribution was relatively homogeneous (Fig 18) with a decreasing trend from the north, where the mean incidence was close to 2 per 100,000 inhabitants, towards the center (average incidence 7 per 100,000 inhabitants) and the South (incidence greater than 9 bites per 100,000 inhabitants). The sex ratio (M/F) was 1.97. The seasonal distribution showed a marked summer increase in snakebites (Fig 18).

### Nicaragua

Notification of snakebites is not available online. The epidemiological data were based on the work by Hansson et al. [44] the source of whom was the Ministry of Health.

According to these authors, there were about 650 snakebites each year (56 per 100,000 inhabitants) and 7 deaths (0.6 per 100,000 inhabitants). The geographical distribution was very heterogeneous, with a higher incidence in the south of the country, largely dependent on altitude, land use and health supply [44].

### Panama

Notification of snakebites in Panama was not available online.

According to the Ministry of Health, the average annual incidence could be 1,900 snake bites (55 bites per 100,000 inhabitants). Valderrama *et al*. [45] mentioned about fifteen deaths per year (0.5 deaths per 100,000 inhabitants). The incidence was highest in the provinces of Darién, Coclé, Los Santos (three provinces in the center of the country) and Veraguas in the east, although in the latter the data were much underestimated. The work by Barahona de Mosca (2003, quoted by Valderrama et al. [45]) showed that people aged 20 to 44 were the most affected (44%), followed by teenagers aged 10–19 (23%), and children 0–9 (18%). In all age groups, males were most often bitten. Highest incidence occurred during the rainy season (from May to November).

### Paraguay

Notification of snakebites has been mandatory since 2008 but was only truly functional from 2009.

Nearly 250 snakebites were reported annually (3.5 per 100,000 population) during the period 2004–2015. Snakebites decreased regularly between 2009 and 2013, and then increased dramatically in 2014 and 2015. However, the general trend of incidence is decreasing (R2 = 0.7319) suggesting that the annual variations are random and risk is reducing. The average number of deaths was 5 per year (0.08 per 100,000 inhabitants).

**Fig 19:**
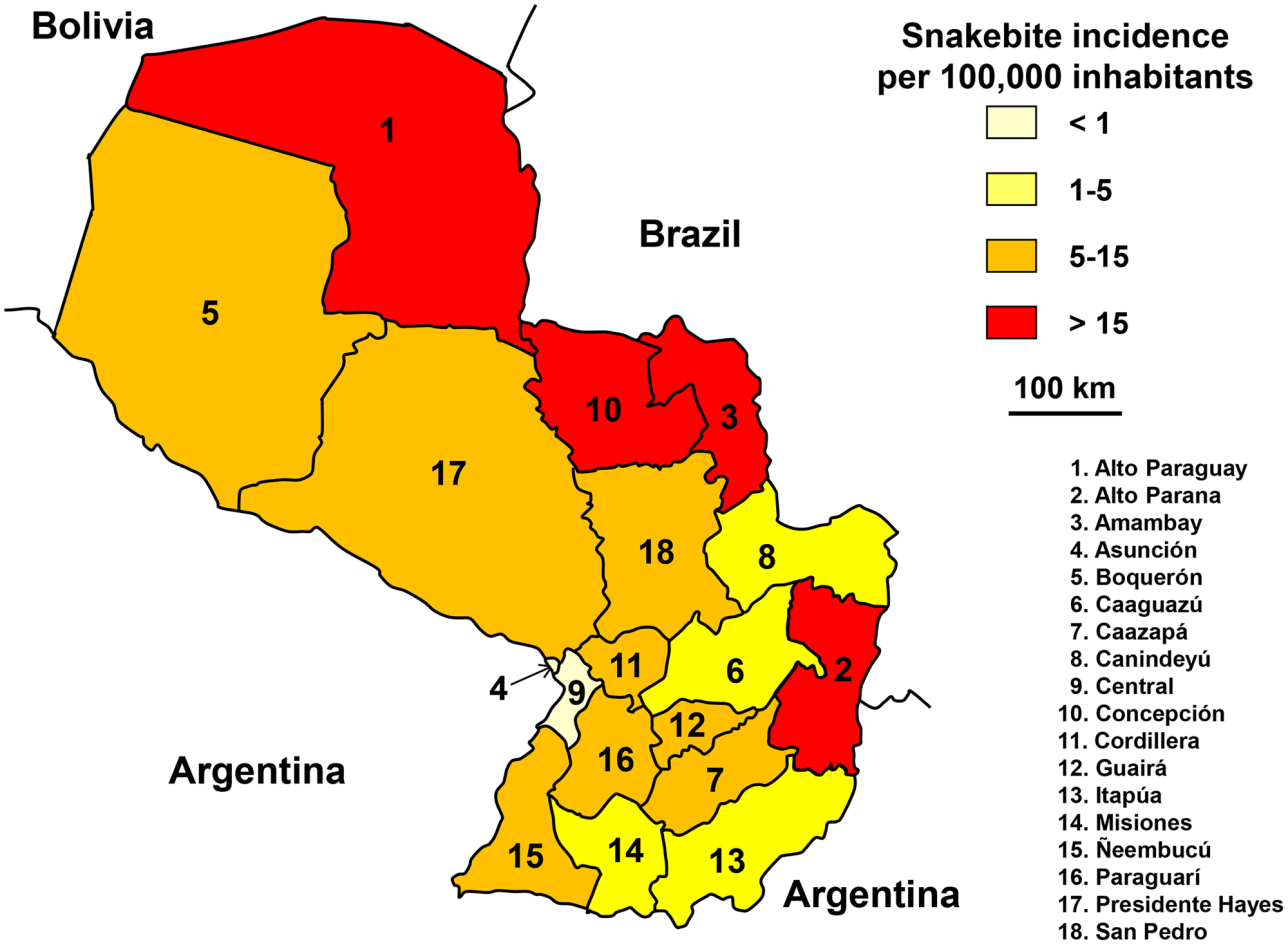
Geographical distribution of incidence of snakebites in Paraguay 2010–2015 (Anonymous, *Paraguay Departements*. Available from https://commons.wikimedia.org/wiki/Atlas_of_Paraguay#/media/File:Paraguay_departements.png [11/28/2016].

The seasonal incidence is relatively constant throughout the year with a slight increase during the rainy season (December to April). The incidence was higher in northern and eastern Paraguay (Fig 19).

### Peru

Notification of snakebites has been available online since 2000.

**Fig 20:**
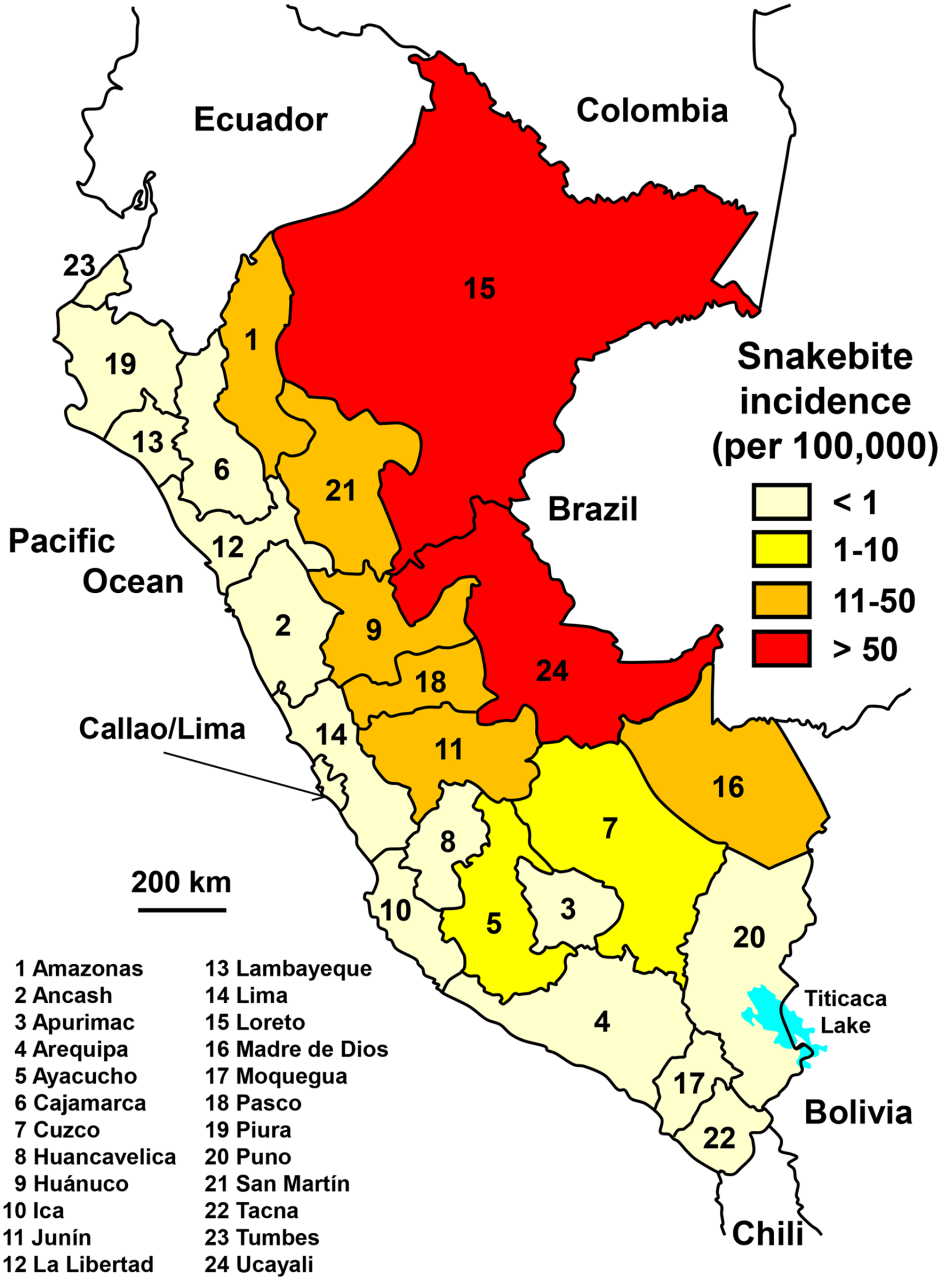
Geographical distribution of incidence of snakebites in Peru 2000–2015 (Guillermo Romero, *Political Map of Peru*. Available from https://commons.wikimedia.org/wiki/Atlas_of_Peru#/media/File:Peru_-_Regions_and_departments_(labeled).svg [08/26/2015].

On average, 2,150 snakebites occurred per year in Peru (7.2 per 100,000 population), resulting in about 10 deaths (0.043 per 100,000 population) during the years 2000–2015. The increase in incidence was significant. However, after a steady increase until 2011, the incidence tends to stabilize or even to decrease slightly in recent years (R^2^ = 0.739). The highest incidence was observed in the Amazon region, while the incidence in the coastal region and the south of the country was low (Fig 20). The seasonal incidence is constant for most of the year with a net decrease in the middle of the dry season (mainly from June to September).

### Saint Lucia

There was no information about Saint Lucia. However, the epidemiological situation should be comparable to that of Martinique, which corresponded to about ten bites per year (6 per 100,000 inhabitants) and one death every 5 to 10 years (0.1 per 100,000 inhabitants). *Bothrops caribbaeus*, a species close to *B*. *lanceolatus*, is endemic to the island [8; 46].

### Suriname

Notification of snakebites was not mandatory in Suriname and no information on envenomation has been found. Based on the situation in French Guiana, the annual number of snakebites can be estimated at 135 (25 per 100,000 inhabitants) and the number of deaths at 5 deaths (0.9 per 100,000 inhabitants).

### Trinidad

Notification was not mandatory in the island of Trinidad for which there was no information on snakebites.

Based on the data collected in coastal Venezuela and Guyana, it can be expected 130 snakebites (10 per 100 000 inhabitants) and 1 to 2 deaths (0.1 per 100 000 inhabitants) each year.

Four poisonous species occur in Trinidad: *Micrurus lemniscatus* and *M*. *circinalis*, both Elapids, and *Bothrops atrox* and *Lachesis muta* that are vipers. *M*. *circinalis* and *M*. *fulvius* are present in some Bocas islands. There is no Elapidae or Viperidae in Tobago [8].

### United States of America

The notification of snakebites in the US was old but hardly available online. Several sources were used and the data were regularly reported in the literature [47–56]. These data were based on notifications from separate systems but were consistent and highly convergent.

**Fig 21:**
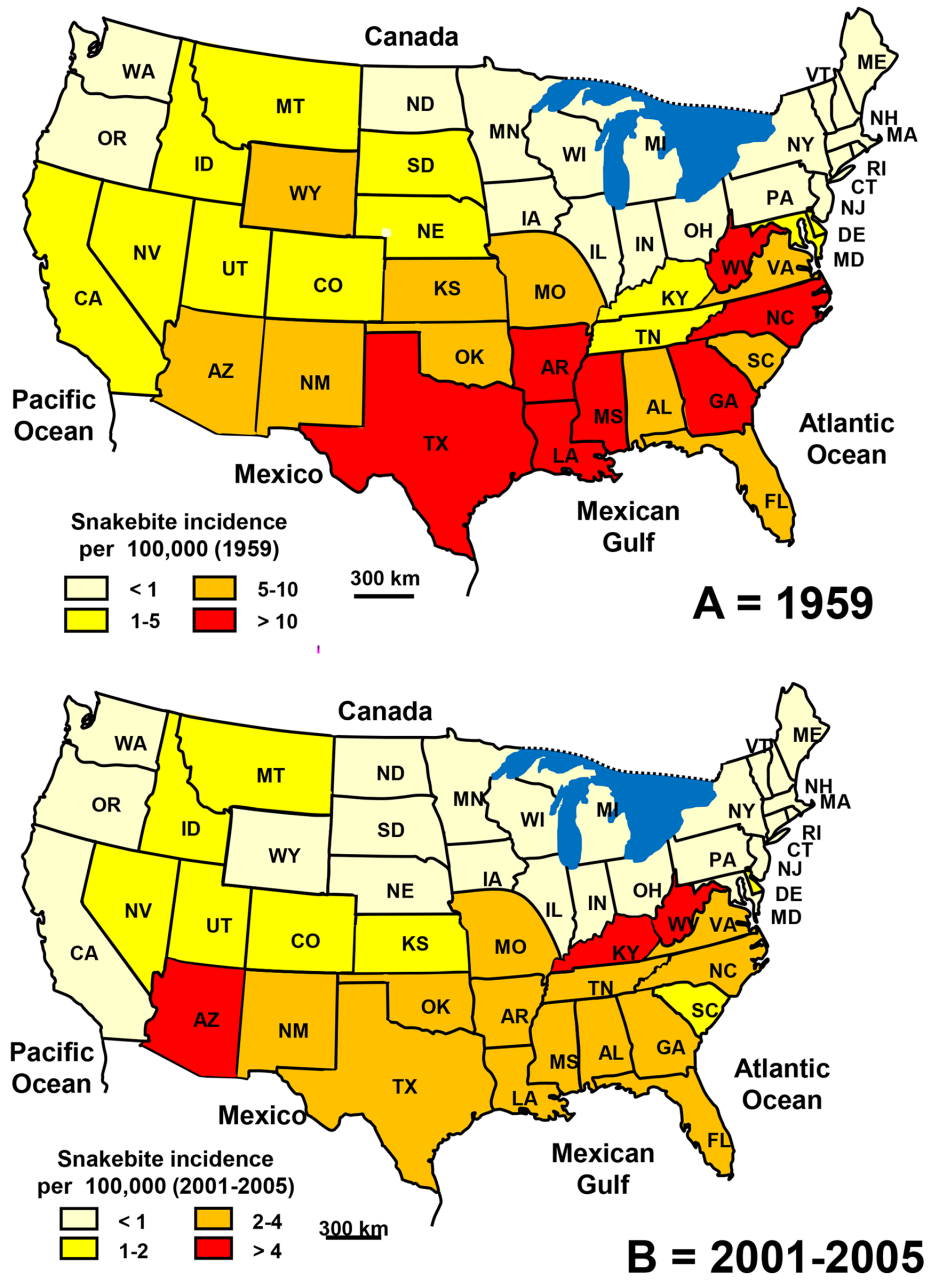
Geographical distribution of incidence of snakebites in the USA. A: data of 1959 from [47]); B: data of 2001–2005 from [53]).

Between the late 1950s and early 2000s, incidence decreased by half (3.6 *versus* 1.7 per 100,000 population) as a result of both the reduction in the number of bites (6,680 in 1959 *versus* 4,735 in 2005) and the increase in population (185 million versus 285 million). The reduction in incidence concerned most of the States, particularly in the southern and eastern US (Fig 21). However, using the National Electronic Injury Surveillance System, Langley *et al*. [56] estimated the number of snakebites (including from non-venomous snakes) to be close to 9,200 on average per year over the period 2001–2010. The number of bites for which the species was identified as venomous would be more than 2,800 per year. Furthermore, Morgan *et al*. [57] reported 97 health deaths from 1979 to 1998, i.e. 4.85 on average per year (0.002 per 100,000 population).

The population at risk was predominantly composed of people whose age is between 10 and 50 years. However, the age-specific incidence showed a peak in teenagers (incidence higher than 5 bites per 100,000 young people aged 10–14 years) and then a steady decrease in adults to about 2 bites per 100,000 Subjects over 65 years of age. The sex ratio (M/F) was 2.7. Most bites occurred from late spring to fall [53].

However, the information provided by the various databases did not detail whether the bites were accidental or illegitimate, the latter probably more frequent in USA, and not seasonal.

### Uruguay

Notification of snakebites was mandatory but data were not available online. However, the Ministry of Health published a summary report on snakebites between 1986 and 2001 and a second on the cases of 2010 and 2011. Despite the lack of information between 2002 and 2009, the incidence was likely to be stable.

**Fig 22:**
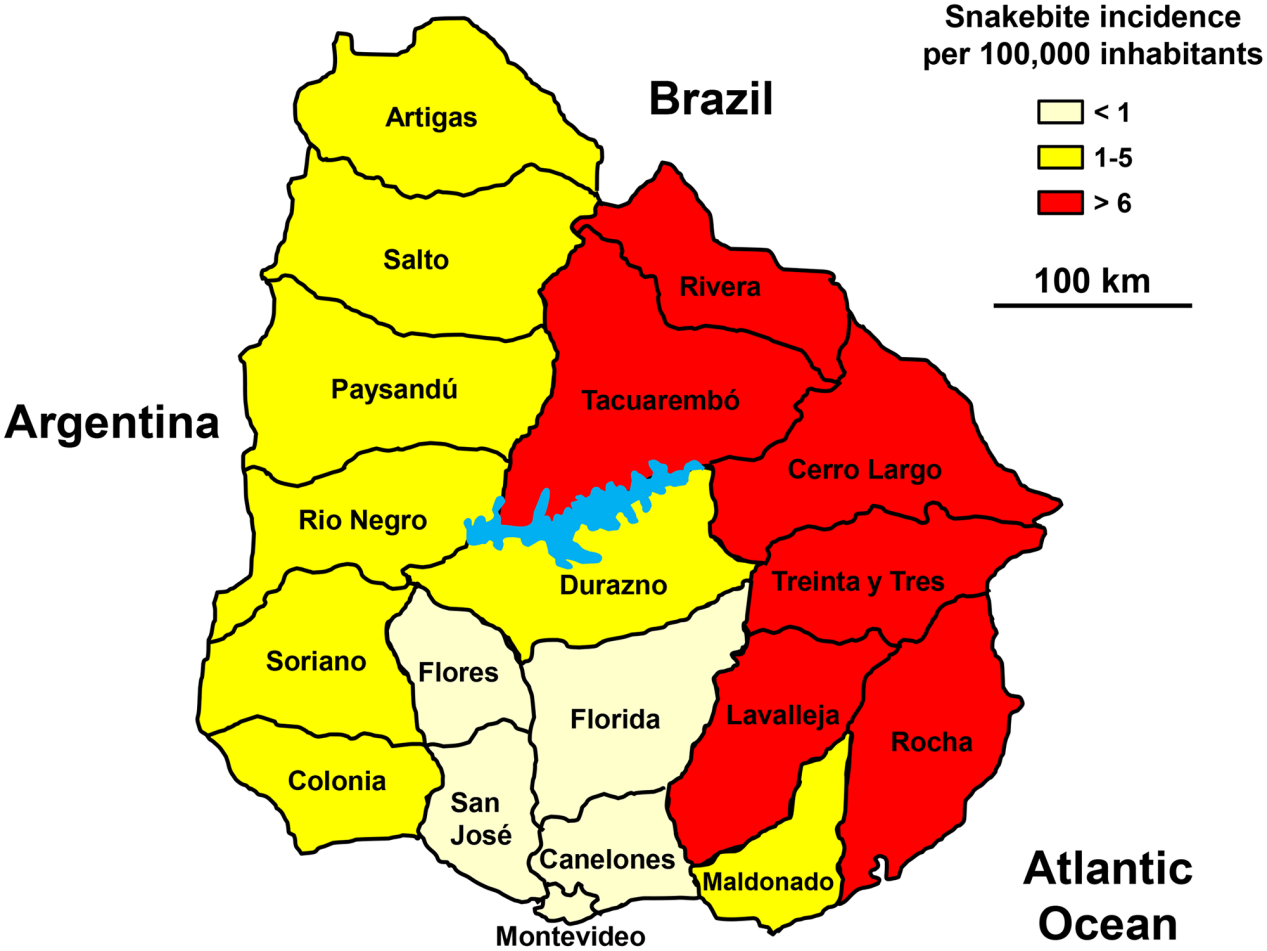
Geographical distribution of incidence of snakebites in Uruguay 1993–2002 (Golbez, *Map of the departments of Uruguay*. Available from https://commons.wikimedia.org/wiki/Atlas_of_Uruguay#/media/File:Uruguay_departments_named.png [07/24/2016].

There are nearly 80 snakebites annually (2.4 per 100,000 population) and 2 deaths (0.033 per 100,000 population). The geographical distribution showed a very high incidence in the eastern part of the country, high in the west and low in the south, especially in the Montevideo region (Fig 22). The age-specific incidence was the highest in young subjects between 15 and 30 years of age. The sex ratio was highly imbalanced in favor of man (M/F = 4.9). The seasonal incidence showed a marked increase in the spring-summer period (October to April) with a peak in March (average cases twice higher than those of other summer months).

### Venezuela

The reporting of snakebite incidence and mortality has been mandatory since 1995 and has been available online since 1996 and 1995 respectively [58].

From 1995–96 to 2012, the average number of snakebites and deaths was 5,700 (20 per 100,000 population) and 32 (0.1 per 100,000 population) a year, respectively. Incidence increased from 1996 to 2006 (R^2^ = 0.7194) and then drastically decreased until 2011 (R^2^ = 0.9576, the last available year. The overall trend is slightly decreasing from 1996 to 2011 (R2 = 0.1507). A possible explanation could be deterioration in the collection of data after 2010 but it is not excluded that changes in economical activities induced a lower snakebite risk.

**Fig 23:**
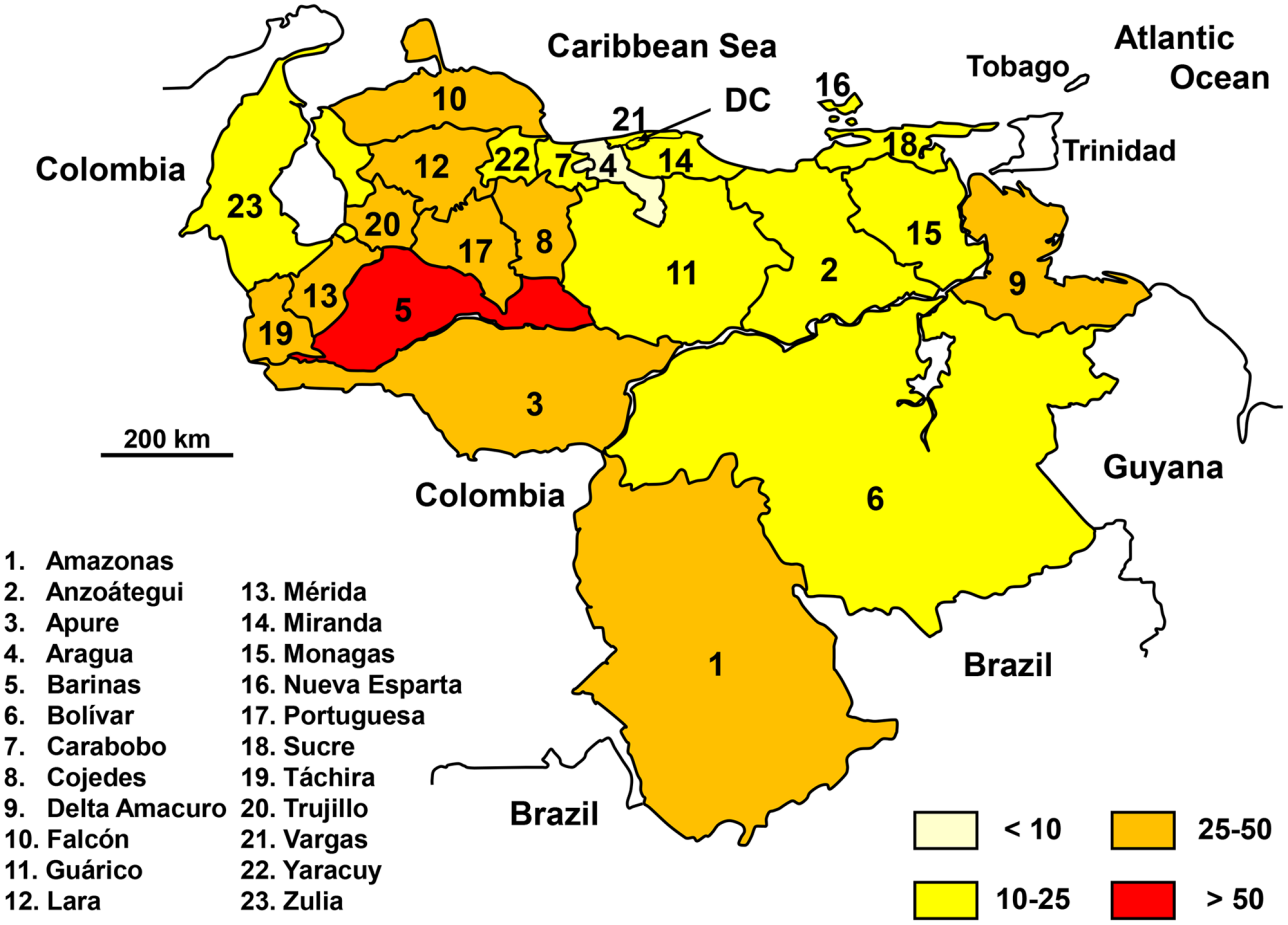
Geographical distribution of incidence of snakebites in Venezuela 1996–2011 (The Photographer, *División Político Territorial de Venezuela*. Available from https://commons.wikimedia.org/wiki/Atlas_of_Venezuela#/media/File:Venezuela_Division_Politica_Territorial.svg [07/28/2016].

The geographical distribution was relatively homogeneous (Fig 23). There is a correlation between the mean incidence of snake bites and population density (R^2^ = 0.6568). Interestingly, the incidence was likely to be underestimated–compared to data from other countries–in some states of the Amazon region, which could be due to either low performances of case reporting system or peculiar treatment seeking behavior by patients, both linked to poor health care offer.

Mortality was relatively constant over time [59]. However, the relative risk of death as a function of age was roughly constant from childhood to adulthood up to 40 years (between 0.05 and 0.09 per 100,000 subjects of each age group) and rose in older people to exceed 0.5 per 100,000 population above 60 years of age.

## Discussion and conclusion

Every year, near 60,000 snakebites (6 per 100,000 inhabitants) are managed by the health services of the Americas. Despite the lack of mortality data in a few countries, most of which are small and poorly populous, the total number of deaths can be estimated at 370 per year (0.04 per 100,000 inhabitants), based on the data from the neighboring countries and risk factors described below.

The previous epidemiological estimates, based mainly on medical and scientific literature, mentioned greater numbers of snakebites: about 115,000 [84,110–140,981] with 2,000 deaths [652–3,466] in the study by Kasturiratne et al. [2] and even 150,000 snakebites of which 5,000 deaths in Chippaux's one [1]. The number of bites did not decreased in the last twenty years (see below), in contrary of deaths. These figures were therefore overestimated, which can be explained by the highly biased epidemiological source of information. Indeed, most authors who publish epidemiological or clinical studies on snakebites report facts upon regions with high incidence—or severity—of envenomation that are often poorly representative [60].

Nevertheless, the general incidence is much lower than in Asia or Africa [1; 2; 61], excluding for particular regions such as the Amazon. However, mortality remains moderate, except in enclosed or poorly equipped areas.

Most of the data collected in this study comes from the Ministries of Health of the concerned countries.

Until now, epidemiological surveys were needed to obtain information that was most often limited geographically according to the constraints and choices of the investigators. Sometimes methodological biases, particularly in site selection, led to approximations or significant errors in the estimation of the incidence or severity of envenomations [62]. For the past decade, mandatory reporting of snakebites resulted in better epidemiological data in most countries of the Americas.

Mandatory reporting of cases allows covering a country as a whole rather than a few sites chosen by the investigators, leading to poorly representative figures. However, data gaps and limitations are still observed resulting from a poor surveillance system. On the one hand, it is expected that over time the data collection will improve and on the other hand the standardization of the questionnaires will make it possible to have more robust, reliable and complete information. For example, useful, often missing data, particularly severity, treatment (brand and dose of antivenom) and clinical outcomes (mortality, sequelae) need to be collected, which is not currently the case in most situations. However, in some countries (Brazil, United States), these data are available, showing that such a goal is feasible.

It is rarely stated whether the notification of snakebites included asymptomatic bites, which is probably the case in most countries. Asymptomatic snakebites may result either from a bite by a non-venomous snake or a venomous one that did not inject venom (dry bite). According to the countries and authors, asymptomatic snakebites represent between 10 and 40%, about one third of which are dry bites [7; 63; 64].

As a consequence, the comparison with the recent literature has been very useful for, a) confirming (or supplementing) the data from other sources and, b) providing additional information, in particular on the clinical severity of envenomations, details on circumstances of the bite or implementation of the treatment.

It was emphasized that the notification was not very precise and reliable, at least variable from one country to another. However, the reporting system improves over the time and, of course, provides a minimal—conservative—incidence of snakebites seen by healthcare institutions from which it can be inferred treatment needs, especially antivenoms. The increase in incidence observed in some countries (Bolivia, Brazil, Colombia, Mexico, Peru, Venezuela) can be attributed to an improvement in data collection, particularly in the early years of its implementation. The stabilization or reversal of the upward trend confirms this. However, environmental (e.g. reduction of snake population) or demographic (population migration to urban centers with low snakebite risk (see below)) causes should not be underestimated. It is notable, for example, that the incidence is often similar on both sides of a border between two neighbor countries—despite likely differences in data collection efficiency -, reflecting a constant figure regarding both risk and population reaction to the snakebite. Actually, administrative policies are different on each side of the border, but populations are often the same on the both sides… It is known, for example, that many patients prefer to use alternative medicine rather than a modern treatment provided by health center. This occurrence is poorly addressed in Latin America, but it probably plays a significant role in underestimating the incidence and possibly severity (mortality) of envenomations. However, some inconsistencies can be explained either by different environmental conditions affecting the risk factors mentioned below, or by significant differences in the quality of the notifications. The report still suffers from inadequacies, resulting in underestimations of snakebite incidence and mortality in some regions of Latin America [44].

The geographical distribution of the incidence was heterogeneous: it was higher in the intertropical region and in developing countries. The incidence depends mainly on environmental and anthropic factors that are detailed below.

The number of deaths appeared to be more difficult to determine due to the lack of notification in several countries. However, these countries are generally sparsely populated regions, which limit the impact on the total result. We proposed here a reasonable estimate for each of these countries at the risk of a trivial error.

Basically, the incidence results from the encounter between a man and a snake. It is therefore legitimate to consider the activities and the presence of the first as well as the behaviors of the latter. It is difficult to explain what affects snakebite incidence because of the complexity of possible causes and their interactions, such as the biology of animal populations composed of many species or the demographics of human populations that are dependent on many social, economic, environmental factors. The coefficient of determination R^2^ indicates the proportion of the variance in the dependent variable that is predictable from the independent variable, i.e. it gives some information about the goodness of fit of a model. The closer R^2^ is to 1, the better the data match the model, but this does not mean the model is relevant.

Incidence tends to grow mechanically as a function of demography although there is a partial offset related to a decrease due to anthropization of the environment which reduces snake populations and/or snake-man contacts. In addition, the proximity of human populations to the natural environment explains a greater frequency of encounters with snakes. As a consequence, snakebites occur usually in rural areas during agricultural activities, especially in developing countries where farming is an important and weakly mechanized economic activity.

Population density was sometimes inversely correlated with the incidence of bites, as in Brazil [20], suggesting that a high human presence limited the development of snake populations. However, other reasons may locally explain the inverse correlation, e.g. when the human population remains large while snakes do not encounter favorable conditions for their development. For instance, the altitude and roughness of the climate appeared to have a negative impact on snake populations as shown in Bolivia or El Salvador, and Canada or Argentina, respectively.

Isolated areas are the most affected, mainly due to lack of good roads linking urban centers and activities of the population performed in precarious conditions (forestry, subsistence agriculture and hunting, among others). These occurrences increase both the likelihood of encounters with snakes and the difficulty of receiving timely medical help. As a consequence, scarcity of health centers is a factor that indirectly influences the incidence of snakebites and directly (and significantly) affects the clinical outcomes of envenomations [33; 44; 65].

The abundance of snakes, especially species that inhabit cultivated or settled areas and sometimes even reproduce there, varies according to climatic (heat and humidity) and environmental (vegetation and landscape) factors that determine food supply, both qualitative and quantitative, and camouflage opportunities [66]. While some species established in natural environments, such as the Amazon rainforest, *e*.*g*. *Bothriopsis taeniata*, are absent or rare in anthropogenic areas, others come near to human settlements and may even grow there [67], at least to some extent. Some species of *Crotalus*, e.g. *C*. *viridis* or *C*. *oreganus* in the USA [68; 69], or *Bothrops*, as *Bothrops asper* in Costa Rica [36], are attracted to anthropogenic areas where they find their food.

Ecological niche modeling (ENM) allows, using appropriate algorithms, to predict the geographic distribution of a species from climatic and environmental data. Yañez-Arenas et al. [70] used the ENM to assess the potential distributions of several species of rattlesnakes in Veracruz and to associate them with a prediction of abundance estimated by the distance from the niche centroid (DNC). These authors found a significant inverse relationship between the snakebites and DNCs of two common vipers (*Crotalus simus* and *Bothrops asper*), partially explaining the variation in the incidence of snakebites. Moreover, the DNCs of the two vipers, combined with the marginalization of human populations, accounted for 3/4 of the variation in incidence. Thus, several factors, environmental, socio-economic and sanitary, contribute to explain the incidence of snakebites.

Populations at risk were very similar in most countries. While children and teenagers constituted an important part of the population, sometimes the majority in developing countries, they were not the mostly bitten. Population at risk was predominantly composed of young men between the ages of 15 and 45, living in rural areas and bitten during agricultural activities. This may explain why bites occur most often during hot (summer) and wet (rainy season) periods, usually at harvest time.

The severity of the envenomation, in particular mortality, is related to the species, but also the size, of the snake responsible for the bite, which determine the composition of the venom and the quantity injected respectively [14; 15; 71]. This explains why some snakebites are asymptomatic, when the snake is not venomous, or when it does not inject its venom [6; 7; 63; 64]. It is more difficult to explain some of the factors identified by Jorge et al. [71] as the season or time of day. This may be due to a particular distribution of species within stands, depending on time and space according to their ecological tropisms.

Age of the patient appeared to be a risk factor, especially at both ends of life, in children and elderly persons–*a priori* more vulnerable [72]. However, as we have seen above, children are not the most exposed.

In addition, the mortality and incidence of complications–most notably the sequelae–depend on the management of snakebites, i.e. the health care system as a whole (number and distribution of health facilities, equipment, access to antivenoms and adequacy of therapeutic protocols, skill of health personnel, etc.). For example, the significant decline in mortality in many countries–particularly in Costa Rica [30–32], Ecuador [34], Mexico [43] and Venezuela [59] while the number of snakebites in these countries remained stable or even increased–can be attributed to better management of snakebites, notably through the improvement of primary health care and access to medical services, including availability of antivenoms.

However, other factors may also affect the mortality and severity of envenomations, such as the availability of health centers and treatment, which may be very irregular, particularly in remote areas where activities of the indigenous population are often very close to nature. The delay in treatment may thus compromise the clinical course of envenomation. Nevertheless, the treatment seeking behavior is complex and many patients, particularly in remote areas, still use traditional medicine. The latter should be associated with modern medicine in order to define relevant recommendations that do not put them into competition but optimize the therapeutic approaches to avoid complications and disabling sequelae as is still often the case.

This study summarized the burden and epidemiological characteristics of snakebites in the American continent. The incidence and severity of envenomation appeared to be lower than previously assessed, although many risk factors have been already known and studied. This work showed the importance of mandatory reporting of snakebites to improve their management, provided that health authorities endorse, analyze and exploit the data.

It therefore seems necessary to continue this effort, improve the case reporting system and take the measures that can be inferred from the obtained analysis of the available information.
